# Antioxidants stimulate BACH1-dependent tumor angiogenesis

**DOI:** 10.1172/JCI169671

**Published:** 2023-10-16

**Authors:** Ting Wang, Yongqiang Dong, Zhiqiang Huang, Guoqing Zhang, Ying Zhao, Haidong Yao, Jianjiang Hu, Elin Tüksammel, Huan Cai, Ning Liang, Xiufeng Xu, Xijie Yang, Sarah Schmidt, Xi Qiao, Susanne Schlisio, Staffan Strömblad, Hong Qian, Changtao Jiang, Eckardt Treuter, Martin O. Bergo

**Affiliations:** 1 Department of Biosciences and Nutrition, Karolinska Institutet, Huddinge, Sweden.; 2 Department of General Surgery and; 3 Department of Thoracic Surgery, The First Affiliated Hospital of Zhengzhou University, Zhengzhou, China.; 4 Department of Laboratory Medicine, Karolinska Institutet, Huddinge, Sweden.; 5 Translational Research Center and Center of Allogeneic Stem Cell Transplantation (CAST), Karolinska University Hospital Huddinge, Stockholm, Sweden.; 6 Center for Hematology and Regenerative Medicine, Department of Medicine Huddinge, Karolinska University Hospital, Huddinge, Sweden.; 7 BGI-Shenzhen, Shenzhen, China.; 8 Department of Oncology-Pathology, Karolinska Institutet, Solna, Sweden.; 9 Department of Physiology and Pathophysiology, School of Basic Medical Sciences, Key Laboratory of Molecular Cardiovascular Science, Ministry of Education, Peking University, Beijing, China.; 10 Center of Basic Medical Research, Institute of Medical Innovation and Research, Peking University Third Hospital, Beijing, China.

**Keywords:** Angiogenesis, Hypoxia, Lung cancer

## Abstract

Lung cancer progression relies on angiogenesis, which is a response to hypoxia typically coordinated by hypoxia-inducible transcription factors (HIFs), but growing evidence indicates that transcriptional programs beyond HIFs control tumor angiogenesis. Here, we show that the redox-sensitive transcription factor BTB and CNC homology 1 (BACH1) controls the transcription of a broad range of angiogenesis genes. BACH1 is stabilized by lowering ROS levels; consequently, angiogenesis gene expression in lung cancer cells, tumor organoids, and xenograft tumors increased substantially following administration of vitamins C and E and *N*-acetylcysteine in a BACH1-dependent fashion under normoxia. Moreover, angiogenesis gene expression increased in endogenous BACH1–overexpressing cells and decreased in *BACH1*-knockout cells in the absence of antioxidants. BACH1 levels also increased upon hypoxia and following administration of prolyl hydroxylase inhibitors in both *HIF1A*-knockout and WT cells. BACH1 was found to be a transcriptional target of HIF1α, but BACH1’s ability to stimulate angiogenesis gene expression was HIF1α independent. Antioxidants increased tumor vascularity in vivo in a BACH1-dependent fashion, and overexpressing BACH1 rendered tumors sensitive to antiangiogenesis therapy. BACH1 expression in tumor sections from patients with lung cancer correlated with angiogenesis gene and protein expression. We conclude that BACH1 is an oxygen- and redox-sensitive angiogenesis transcription factor.

## Graphical abstract

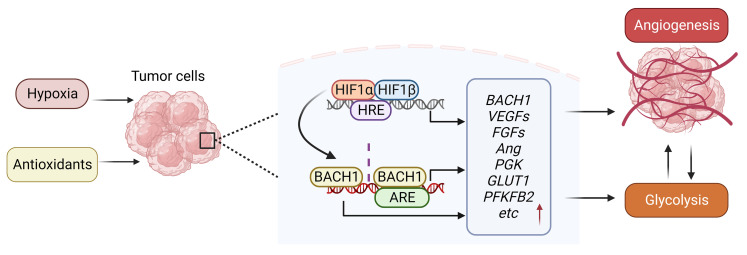

## Introduction

Lung tumor growth and metastasis requires angiogenesis — the formation of new blood vessels (1, 2). Angiogenesis is typically triggered by hypoxia, which stabilizes hypoxia-inducible transcription factors (HIFs) including HIF1α and HIF2α, which mediate the transcription of genes including VEGFs, their receptors (VEGFRs), neuropilin coreceptors (NRPs), EGFs, and angiopoietins (ANGs). Antiangiogenic drugs targeting these proteins and downstream signaling partners have been developed and approved by the FDA for use in combination with conventional chemotherapy in patients with non–small cell lung cancer (NSCLC) (3–7). However, the effects are varied and associated with significant side effects (8, 9). There is also growing evidence that angiogenesis is controlled by transcriptional mechanisms beyond HIFs (10, 11). Thus, identifying new proteins and mechanisms that control tumor angiogenesis as well as tumor biomarkers that are associated with heightened sensitivity to antiangiogenic drugs is a worthwhile effort.

BTB and CNC homology 1 (BACH1) is a redox-sensitive transcription factor that binds antioxidant response elements and is known for its ability to suppress heme oxygenase 1 transcription (12). During oxidative stress, heme released from heme-containing proteins stimulates BACH1 degradation via the ubiquitin ligase FBXO22 (13). Recent studies revealed that lowering oxidative stress in lung cancer cells with *N*-acetylcysteine (NAC) or vitamin E (VitE), or by activating NRF2 reduces ROS and heme levels, which stabilizes BACH1 and activates the transcription of prometastatic genes including *HK2* and *GAPDH* (14). Antioxidants thus stimulate aerobic glycolysis and increase local and distant lung cancer metastasis in a BACH1-dependent fashion (14). Antioxidants also accelerate malignant melanoma metastasis (15, 16).

Glycolysis is often linked with angiogenesis in tumor progression. HIF1α stimulates angiogenesis, which provides oxygen and nutrients to the tumor and upregulates its ability to take up glucose for glycolysis, which in turn provides energy for further angiogenesis and cell proliferation (17). The outcome of BACH1 stabilization following antioxidant administration — i.e., glycolysis and tumor progression — is like that of HIF1α, which is stabilized following hypoxia. We therefore wondered whether BACH1 might also stimulate angiogenesis in response to antioxidants and hypoxia. On one hand, this idea seems counterintuitive, as BACH1 has been suggested to repress angiogenesis (18–20) and VitC was found to reduce HIF1α levels and target gene expression in some cancer cell lines (21). On the other hand, BACH1 was found to be associated with VEGFC expression and angio- and lymphangiogenesis in zebrafish (22) and to be increased during hypoxia (23, 24). In this study, we used human and mouse lung cancer cell lines, tumor organoids, and endogenous and xenograft mouse models to address this issue.

## Results

### BACH1 controls the expression of angiogenesis genes in lung tumor organoids and spheroids under normoxia.

**Figure 1:**
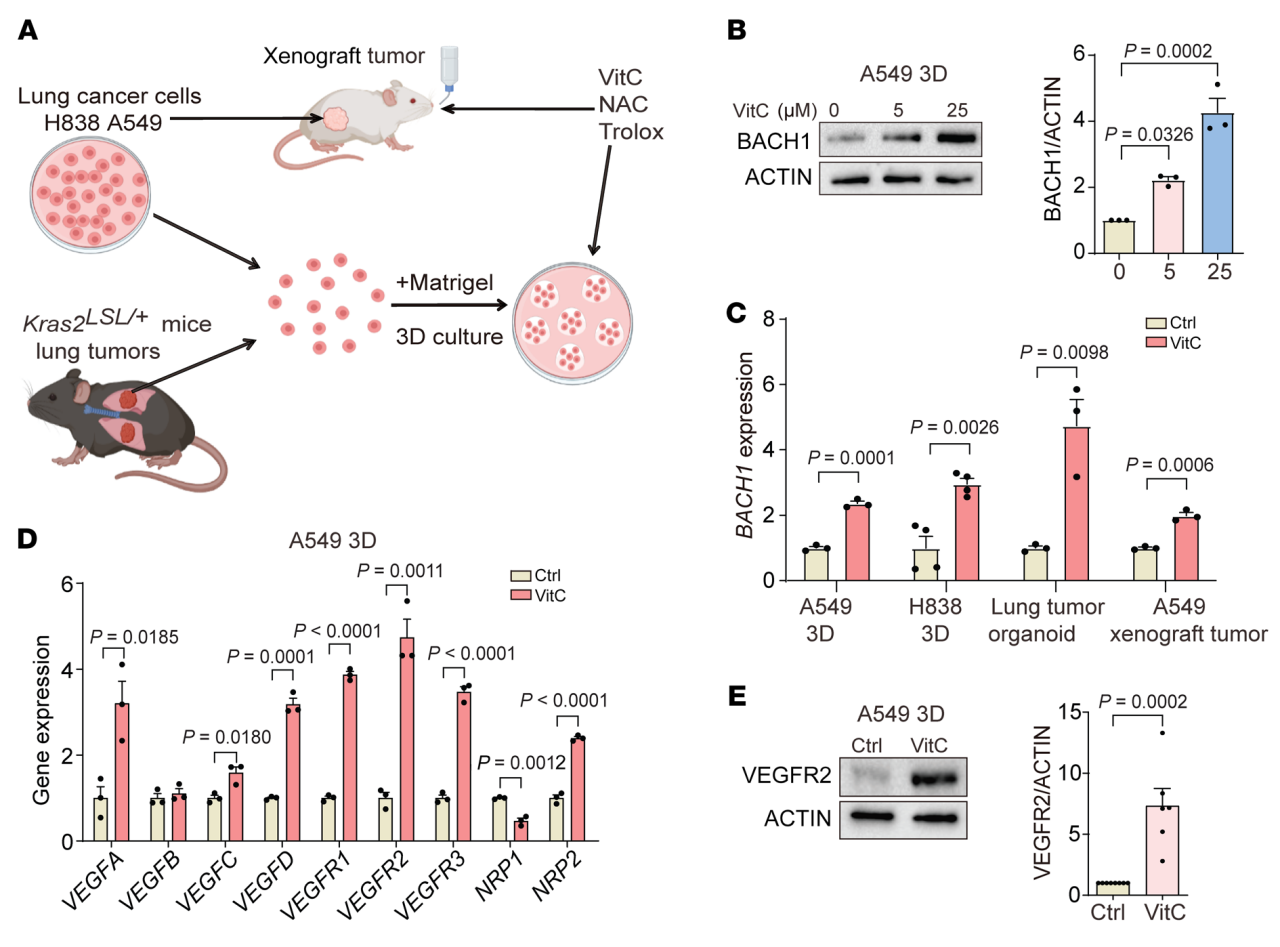
Antioxidants stabilize BACH1 and induce angiogenesis gene expression in NSCLC organoids and tumors by upregulating BACH1 expression. (**A**) Experimental design. (**B**) BACH1 protein levels in spheroids incubated for 7 days with 25 μM VitC and BACH1 levels by densitometry (*n* = 3 experiments). (**C**) Reverse transcription quantitative PCR (RT-qPCR) of *BACH1* in A549 and H838 spheroids , lung tumor organoids incubated for 7 days with 25 μM VitC, and A549 xenograft tumors from mice administered VitC (3.47 g/L) in the drinking water for 7 weeks (*n* = 3 experiments). (**D**) RT-qPCR of angiogenesis genes in spheroids incubated with 25 μM VitC for 7 days (*n* = 3 experiments). (**E**) VEGFR2 protein levels in spheroids incubated with 25 μM VitC for 7 days and VEGFR2 levels determined by densitometry (*n* = 6 experiments). Ctrl, control. Data indicate the mean ± SEM. Statistical significance was determined by 2-tailed, unpaired Student’s *t* test (**C**–**E**) and 1-way ANOVA with Tukey’s post hoc test for multiple comparisons (**B**).

To explore the role of BACH1 in angiogenesis, we first established 3D cultures of the human lung cancer cell lines A549 and H838; tumor organoids from mice with KRAS^G12D^-induced lung cancer; and xenograft tumors from *NSG* mice injected s.c. with A549 cells (Figures 1A and Supplemental Figure 1, A–C; supplemental material available online with this article; https://doi.org/10.1172/JCI169671DS1). Consistent with previous studies (14), administration of VitC, NAC, and Trolox increased BACH1 protein levels; *BACH1* mRNA levels also increased (Figure 1, B and C, and Supplemental Figure 1, D–H). Moreover, the compounds were found to function as antioxidants, as H_2_O_2_ levels decreased and ratios of glutathione (GSH) and glutathione disulfide (GSSG) increased (Supplemental Figure 1I).

**Figure 2:**
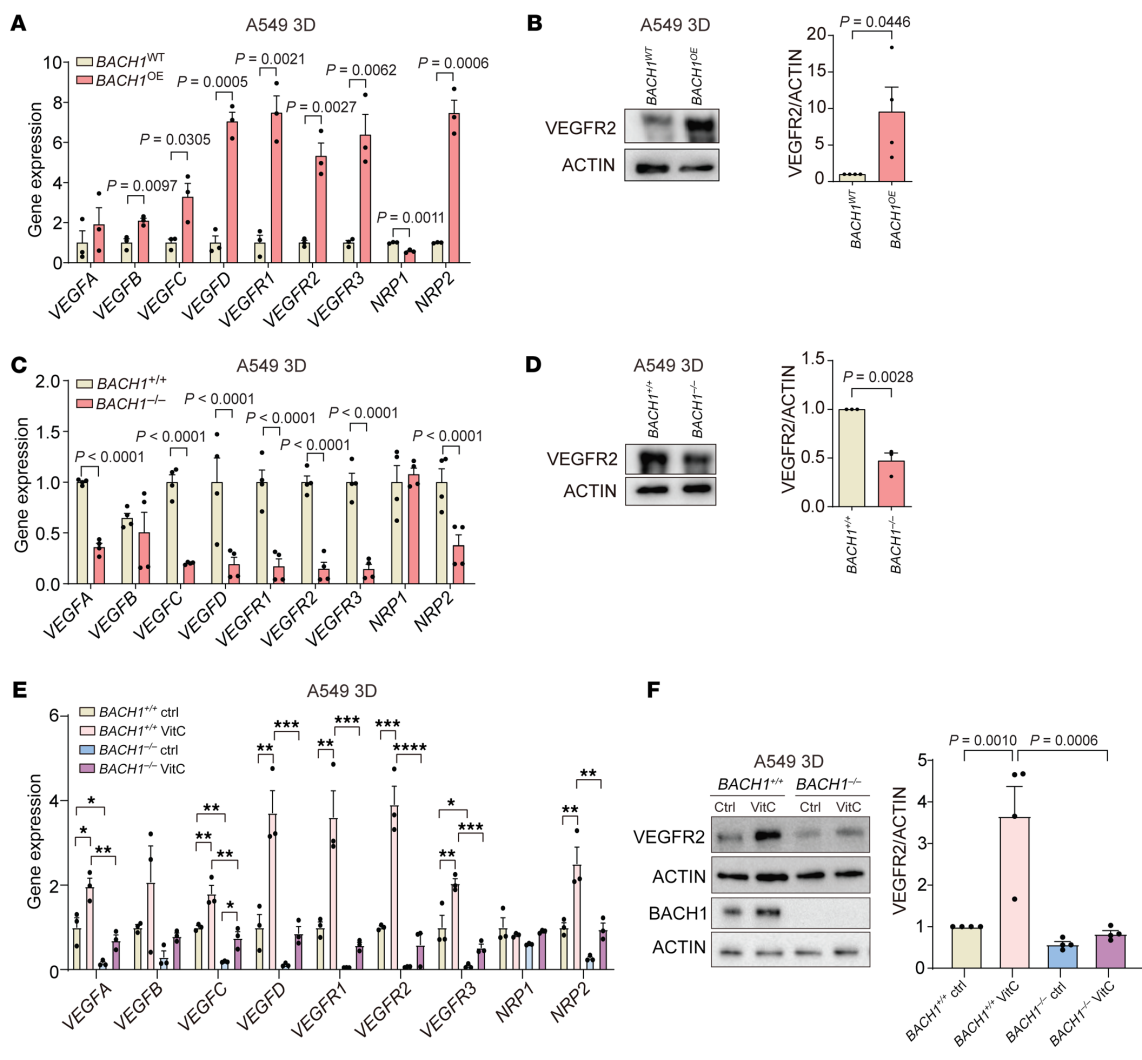
BACH1 controls the expression of angiogenesis genes under normoxia. (**A**) RT-qPCR of angiogenesis genes in *BACH1*^OE^ and *BACH1*^WT^ spheroids under normoxia (*n* = 3 experiments). (**B**) VEGFR2 protein levels in *BACH1*^OE^ and *BACH1*^WT^ spheroids and VEGFR2 levels by densitometry (*n* = 4 experiments). (**C**) RT-qPCR of angiogenesis genes in *BACH1^+/+^* and *BACH1^–/–^* spheroids under normoxia (*n* = 4 experiments). (**D**) VEGFR2 protein levels in *BACH1^+/+^* and *BACH1^–/–^* spheroids and VEGFR2 levels by densitometry (*n* = 3 experiments). (**E**) RT-qPCR of angiogenesis genes in *BACH1^+/+^* and *BACH1^–/–^* spheroids incubated for 7 days with 25 μM VitC or vehicle (Ctrl) (*n* = 3 experiments). (**F**) Top, VEGFR2 and BACH1 protein levels in *BACH1^+/+^* and *BACH1^–/–^* spheroids incubated for 7 days with 25 μM VitC and VEGFR2 protein levels by densitometry (*n* = 4 experiments). Data indicate the mean ± SEM. *P < 0.05, **P < 0.01, ***P < 0.005, and ****P < 0.001, by 2-tailed, unpaired Student’s *t* test (**A**–**D**) and 1-way ANOVA with Tukey’s post hoc test for multiple comparisons (**E** and **F**).

VitC, NAC, and Trolox administration substantially increased the expression of angiogenesis genes including VEGFs, VEGF receptors, and NRPs in the 3D and organoid cultures and xenograft tumors; protein levels of 2 selected genes, VEGFR2 and NRP2, increased concomitantly (Figure 1, D and E, and Supplemental Figure 2, A–J). To determine whether BACH1 is functionally involved in angiogenesis gene and protein expression — in the absence of antioxidants — we used CRISPR/Cas9 strategies to increase and decrease endogenous *BACH1* expression in A549 cells (14). We found that cells with high *BACH1* expression (*BACH1* overexpression [*BACH1*^OE^]) exhibited increased the expression of most of the tested angiogenesis genes and higher VEGFR2 and NRP2 protein levels, whereas cells with low *BACH1* expression (*BACH1^–/–^*) exhibited decreased angiogenesis gene and protein expression (Figure 2, A–D, and Supplemental Figure 2, K–L). Antioxidant administration and BACH1 manipulations caused changes in glycolysis similar to those for angiogenesis gene expression (Supplemental Figure 3, A–F). The ability of VitC to increase angiogenesis gene expression and VEGFR2 protein levels was substantially lower in *BACH1^–/–^* than in *BACH1^+/+^* cells, suggesting that BACH1 mediated antioxidant-induced angiogenesis gene expression. We observed similar results with NAC and Trolox and with glycolysis gene expression (Figure 2, E and F, and Supplemental Figure 4, A–I).

### BACH1-mediated expression of angiogenesis and glycolysis genes correlates with BACH1-dependent epigenetic changes at promoter regions.

**Figure 3:**
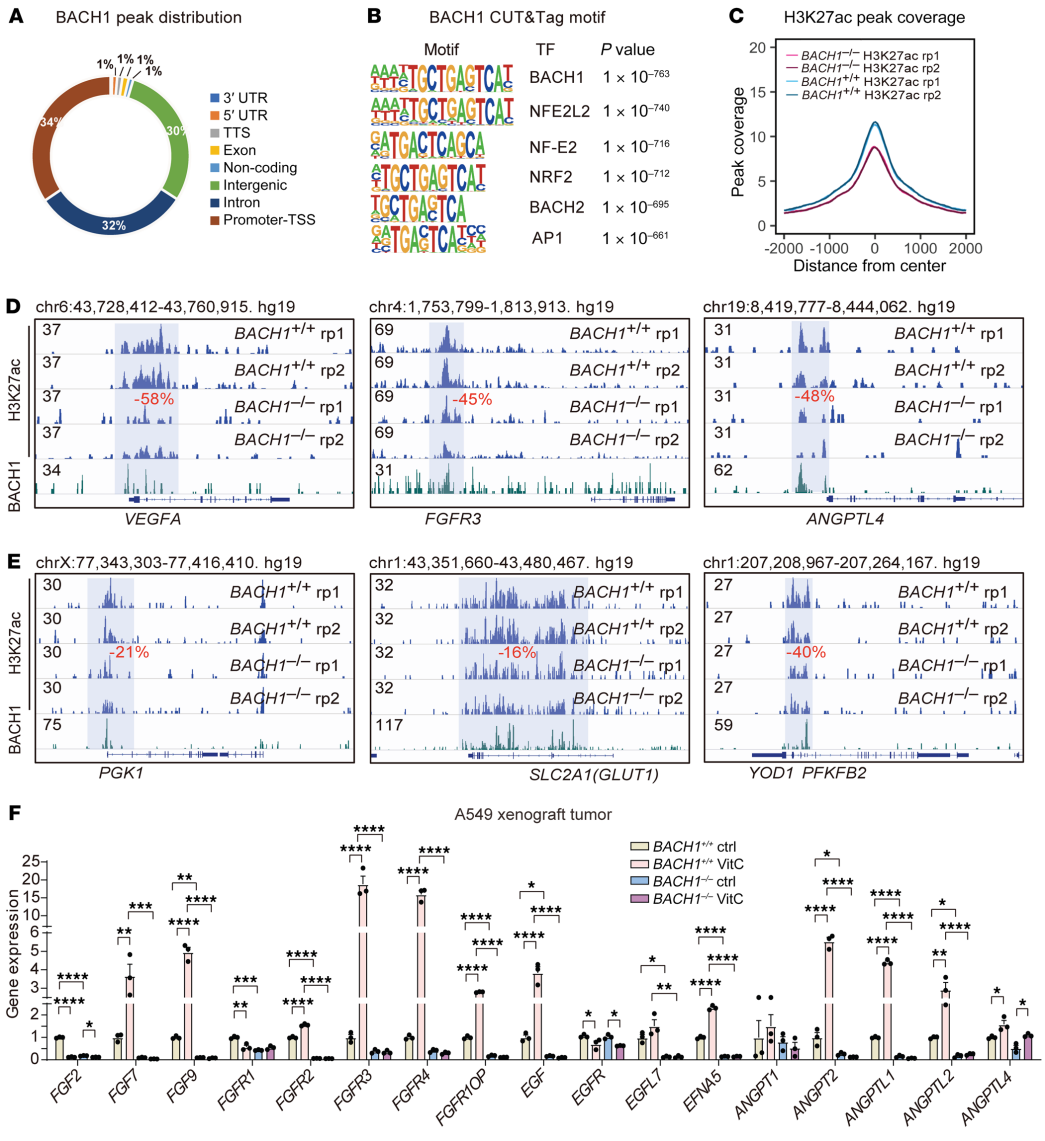
BACH1-mediated expression of angiogenesis and glycolysis genes correlates with BACH1-dependent epigenetic changes at promoter regions. (**A**) Genome-wide profiling of BACH1 chromatin enrichment in A549 spheroids using CUT&Tag. (**B**) Transcription factor DNA-binding motif analysis of BACH1 CUT&Tag peaks. (**C**) Genome-wide plot of H3K27ac peak density in *BACH1^+/+^* and *BACH1^–/–^* A549 spheroids; note that the 2 lines for each genotype replicate (rp1/rp2) overlap. (**D** and **E**) Integrative Genomics Viewer (IGV) tracks showing H3K27ac levels at the indicated angiogenesis (**D**) and glycolysis (**E**) gene loci in *BACH1^+/+^* and *BACH1^–/–^* A549 spheroids. BACH1 peaks are shown at the bottom to indicate overlap with H3K27ac-marked regions. Regions with significant H3K27ac changes in *BACH1^–/–^* compared with *BACH1^+/+^* A549 spheroids are highlighted in blue; the percentage of change is indicated in red. (**F**) RT-qPCR of the expression of a broader set of angiogenesis-related genes in tumors from mice engrafted with *BACH1^+/+^* and *BACH1^–/–^* A549 lung cancer cells. The mice were given VitC (3.47 g/L) or normal drinking water for 7 weeks (*n* = 3 experiments). Data indicate the mean ± SEM. *P < 0.05, **P < 0.01, ***P < 0.005, and ****P < 0.001, by 1-way ANOVA with Tukey’s post hoc test for multiple comparisons (**F**).

We next applied Cleavage Under Targets and Tagmentation (CUT&Tag) to analyze the genome-wide chromatin binding of BACH1, along with H3K27ac marking of transcriptionally active enhancers and promoters. We found that BACH1 bound primarily to promoter regions near transcriptional start sites and to candidate enhancers within intergenic regions and introns (Figure 3A). The BACH1 CUT&Tag peaks were specific, as they highly enriched the BACH1 DNA binding motif, which is also recognized by NFE2, NRF2, BACH2, and AP1 (Figure 3B). Knockout of *BACH1* reduced H3K27ac levels, both genome-wide and at promoters and enhancers of angiogenesis and glycolysis genes (Figure 3, C–E, and Supplemental Figure 5, A–C), suggesting that BACH1 acts directly as a transcriptional activator at these regulatory elements (Supplemental Table 1). Further analyses revealed that basal and VitC-induced expression of members of an extended family of angiogenesis and glycolysis genes was abolished following *BACH1* knockout (Figure 3F).

### BACH1 expression under normoxia and hypoxia is HIF1α dependent, but BACH1 is sufficient for the stimulation of angiogenesis gene expression in HIF1Α-deficient cells.

**Figure 4:**
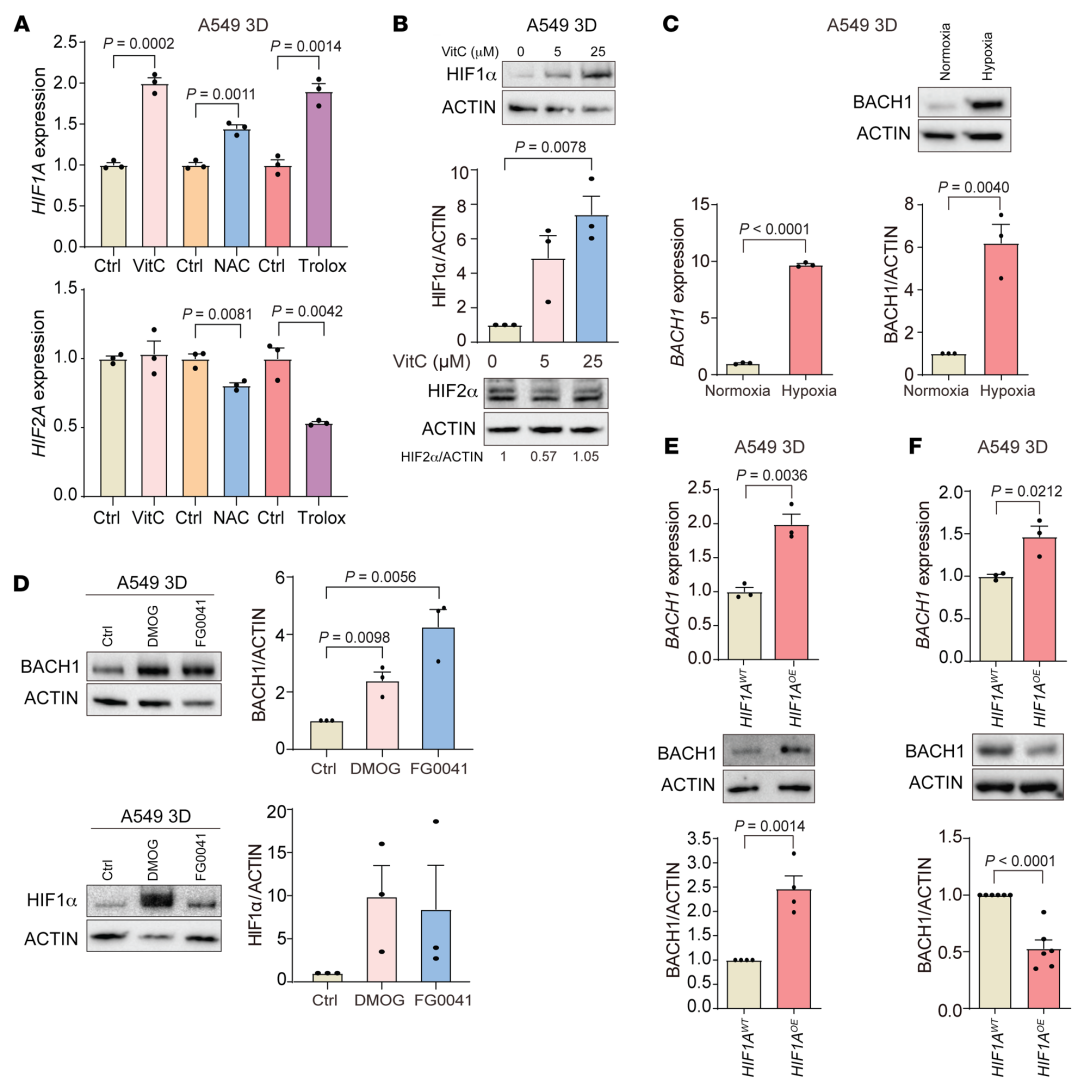
BACH1 expression under normoxia and hypoxia is HIF1α dependent. (**A**) RT-qPCR of *HIF1A* and *HIF2A* in spheroids incubated for 7 days with antioxidants under normoxia (*n* = 3 experiments). (**B**) Top: HIF1α levels in spheroids incubated with VitC by Western blotting. Middle: HIF1α levels by densitometry (*n* = 3 experiments). Bottom: HIF2α protein levels by Western blotting. (**C**) RT-qPCR of *BACH1* expression in spheroids under normoxia (21% O_2_) and hypoxia (1% O_2_) (*n* = 3 experiments), BACH1 protein levels by Western blotting, and BACH1 levels by densitometry (*n* = 3 experiments. (**D**) Left top: BACH1 protein levels by Western blotting in spheroids incubated for 16 hours with prolyl hydroxylase inhibitors. Right top: BACH1 levels by densitometry (*n* = 3 experiments). Left bottom: HIF1α protein levels by Western blotting. Right bottom: HIF1α levels by densitometry (*n* = 3 experiments). (**E**) Top: RT-qPCR of *BACH1* expression in HIF1α-overexpressing (*HIF1A*^OE^) and control (*HIF1A*^WT^) spheroids under normoxia (*n* = 3 experiments). Middle: BACH1 protein levels by Western blotting. Bottom: BACH1 levels by densitometry (*n* = 4 experiments). (**F**) Experiments similar to those in **E** using HIF2α-overexpressing (*HIF2A*^OE^) and control (*HIF2A*^WT^) spheroids (*n* = 6 experiments). Data indicate the mean ± SEM. *P* values were determined by 2-tailed, unpaired Student’s *t* test (**A**, **C**, **E**, and **F**) and 1-way ANOVA with Tukey’s post hoc test for multiple comparisons (**B** and **D**).

HIF1α stabilization during hypoxia stimulates angiogenesis and glycolysis gene expression, so we therefore asked whether increased HIF1α gene or protein levels accompany antioxidant-induced angiogenesis and glycolysis gene expression during normoxia. VitC, NAC, and Trolox increased *HIF1A*, but not *HIF2A*, gene expression in A549 spheroids (Figure 4A). Moreover, the antioxidants dose-dependently increased HIF1α protein levels in A549 and H838 spheroids and lung tumor organoids but had little effect on HIF2α levels (Figure 4B and Supplemental Figure 6, A–C). BACH1 gene expression and protein levels increased during hypoxia (Figure 4C and Supplemental Figure 7A). To explore the mechanism underlying this regulation, we incubated A549 spheroids with the prolyl hydroxylase inhibitors dimethyloxaloylglycine (DMOG) and FG0041 (25) and found that they increased BACH1 protein levels during normoxia in the absence of other stimuli (Figure 4D); control experiments revealed that HIF1α protein levels increased as expected in response to the 2 compounds (Figure 4D).

**Figure 5:**
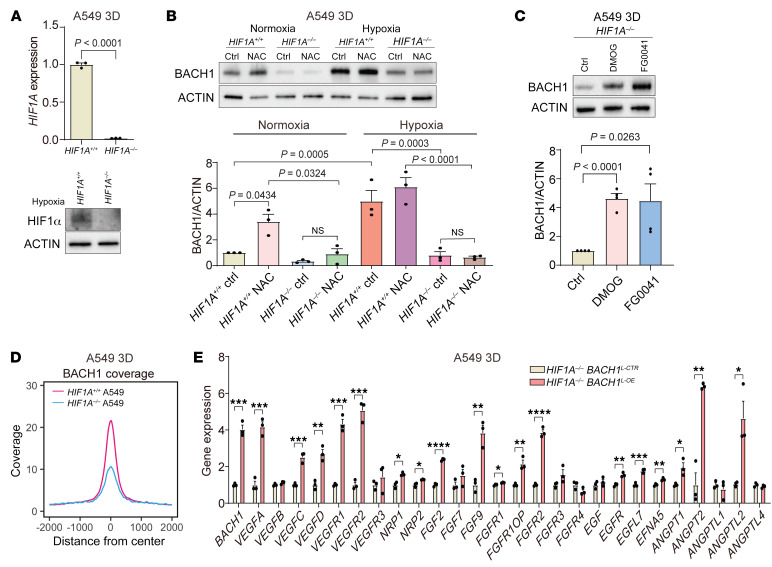
BACH1 increases angiogenesis gene expression in *HIF1A*-deficient lung cancer cells. (**A**) *HIF1A*-knockout validation with RT-qPCR and Western blotting. (**B**) BACH1 protein levels by Western blotting in *HIF1A^–/–^* and *HIF1A^+/+^* spheroids under normoxia and hypoxia and BACH1 levels by densitometry (*n* = 3 experiments). (**C**) BACH1 protein levels by Western blotting in *HIF1A^–/–^* spheroids incubated for 16 hours with prolyl hydroxylase inhibitors and BACH1 levels by densitometry (*n* = 4 experiments). (**D**) Genome-wide BACH1 CUT&Tag peak density plot of *HIF1A^+/+^* and *HIF1A^–/–^* spheroids. (**E**) RT-qPCR of *BACH1* and angiogenesis genes in *HIF1A^–/–^* spheroids with lentiviral BACH1 overexpression (*BACH1*^L-OE^) and controls (*BACH1*^L-CTR^) (*n* = 3 experiments). Data indicate the mean ± SEM. *P < 0.05, **P < 0.01, ***P < 0.005, and ****P < 0.001, by 2-tailed, unpaired Student’s *t* test (**A** and **E**) and 1-way ANOVA with Tukey’s post hoc test for multiple comparisons (**B** and **C**).

Overexpression of *HIF1A*, but not *HIF2A*, in A549 spheroids also increased BACH1 gene and protein levels under normoxia (Figure 4, E and F, and Supplemental Figure 7, B and C). Conversely, basal BACH1 protein levels were markedly lower in *HIF1A^–/–^* than in control *HIF1A^+/+^* A549 spheroids under normoxia, and the ability of antioxidants to increase BACH1 levels under normoxia was abolished in the *HIF1A^–/–^* cells (Figure 5, A and B, and Supplemental Figure 7, D and E). As in the earlier experiments, BACH1 levels increased upon hypoxia — to levels exceeding those observed with antioxidants under normoxia, and BACH1 levels also tended to increase in *HIF1A^–/–^* cells upon hypoxia (Figure 5B, lanes 3 and 7, and Supplemental Figure 7, D and E). Incubation of *HIF1A^–/–^* cells with DMOG or FG0041 increased BACH1 protein levels to an extent similar to that detected in *HIF1A^+/+^* cells (Figure 5C, compare with Figure 4D). Control experiments revealed that reexpression of exogenous *HIF1A* in *HIF1A^–/–^* cells increased both basal and NAC-induced BACH1 levels (Supplemental Figure 7, F and G). We conclude that HIF1α sustained basal BACH1 levels and mediated antioxidant-induced increases in BACH1 levels during normoxia, and that BACH1 gene expression and protein levels increased upon hypoxia in a HIF1α-dependent fashion. The finding that BACH1 protein levels also increased in *HIF1A^–/–^* cells under hypoxia and in response to hypoxia-mimetic drugs suggests a HIF1α-independent, prolyl hydroxylase–dependent regulation of BACH1.

CUT&Tag analyses revealed that the genome-wide BACH1 chromatin occupancy was lower in *HIF1A^–/–^* than in *HIF1A^+/+^* cells, consistent with the downregulation of *BACH1* expression in *HIF1A^–/–^* cells (Figure 5D). However, overexpression of BACH1 in *HIF1A^–/–^* cells markedly increased the expression of a broad range of angiogenesis and glycolysis genes, demonstrating HIF1α-independent regulation by BACH1 (Figure 5E). CUT&Tag experiments with cells under hypoxia demonstrated increased HIF1α chromatin occupancy both genome-wide (Supplemental Figure 8A) and at individual gene loci including in the *BACH1* gene itself (Supplemental Figure 8, C–F). Transcription factor motif analysis showed enrichment of binding sites for HIF1/2α, Kruppel-like family 1 (KLF1), and BACH1 (Supplemental Figure 8B). These results demonstrate that *BACH1* is a transcriptional target of HIF1α but also that BACH1 can stimulate HIF1α-independent angiogenesis and glycolysis gene expression.

### BACH1 expression correlates with angiogenesis gene and protein expression in human NSCLC tumors and increases tumor vascularity and the response to anti-VEGF therapy in xenograft tumors.

**Figure 6:**
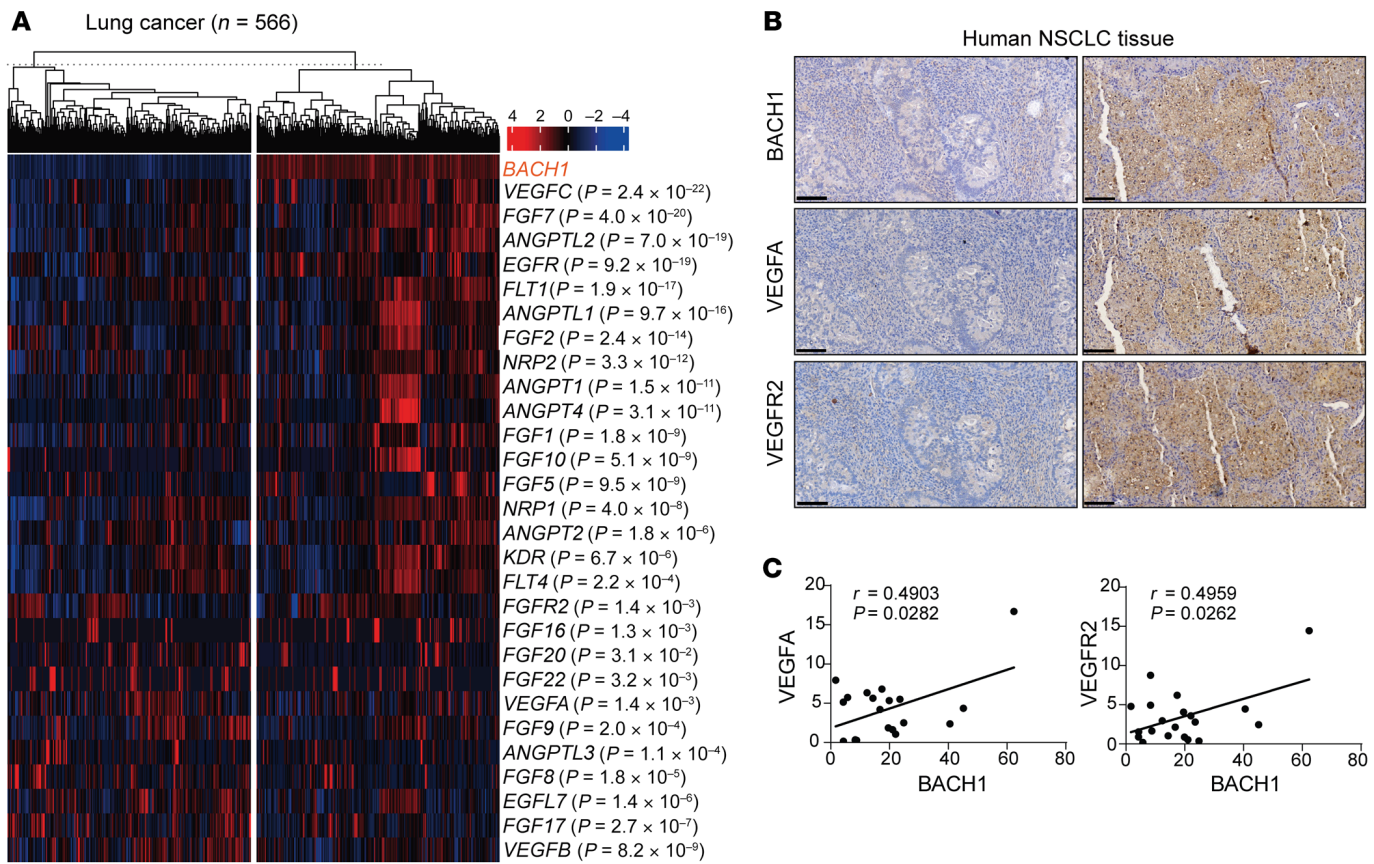
BACH1 expression correlates with angiogenesis gene and protein expression in human NSCLC samples. (**A**) Heatmap showing TCGA lung cancer cases with low (left) and high (right) *BACH1* expression. Angiogenic genes whose expression differed significantly between the 2 groups are listed on the right along with the *P* value for the correlation with *BACH1* expression. (**B**) Representative immunohistochemical staining for BACH1, VEGFA, and VEGFR2 in consecutive sections of tumors from patients with KRAS-mutant NSCLC. Tumor sections with low BACH1 expression (left); tumor section with high BACH1 expression (right). Original magnification, ×20. Scale bars: 100 μm. (**C**) Comparisons of VEGFA and VEGFR2 expression with BACH1 protein expression in human NSCLC tumor sections (*n* = 20). Data were analyzed using Pearson’s correlation test (**C**).

Analyses of The Cancer Genome Atlas (TCGA) data revealed that *BACH1* expression in lung cancers correlates with the expression of a broad range of angiogenesis and glycolysis genes; we observed similar results in breast and kidney cancer cohorts (Figures 6A and Supplemental Figure 9, A and B). Immunohistochemical analyses of tumor sections from patients with KRAS-mutant NSCLC showed correlations between BACH1 and VEGFA and BACH1 and VEGFR2 (Figure 6, B and C, and Supplemental Table 2).

**Figure 7:**
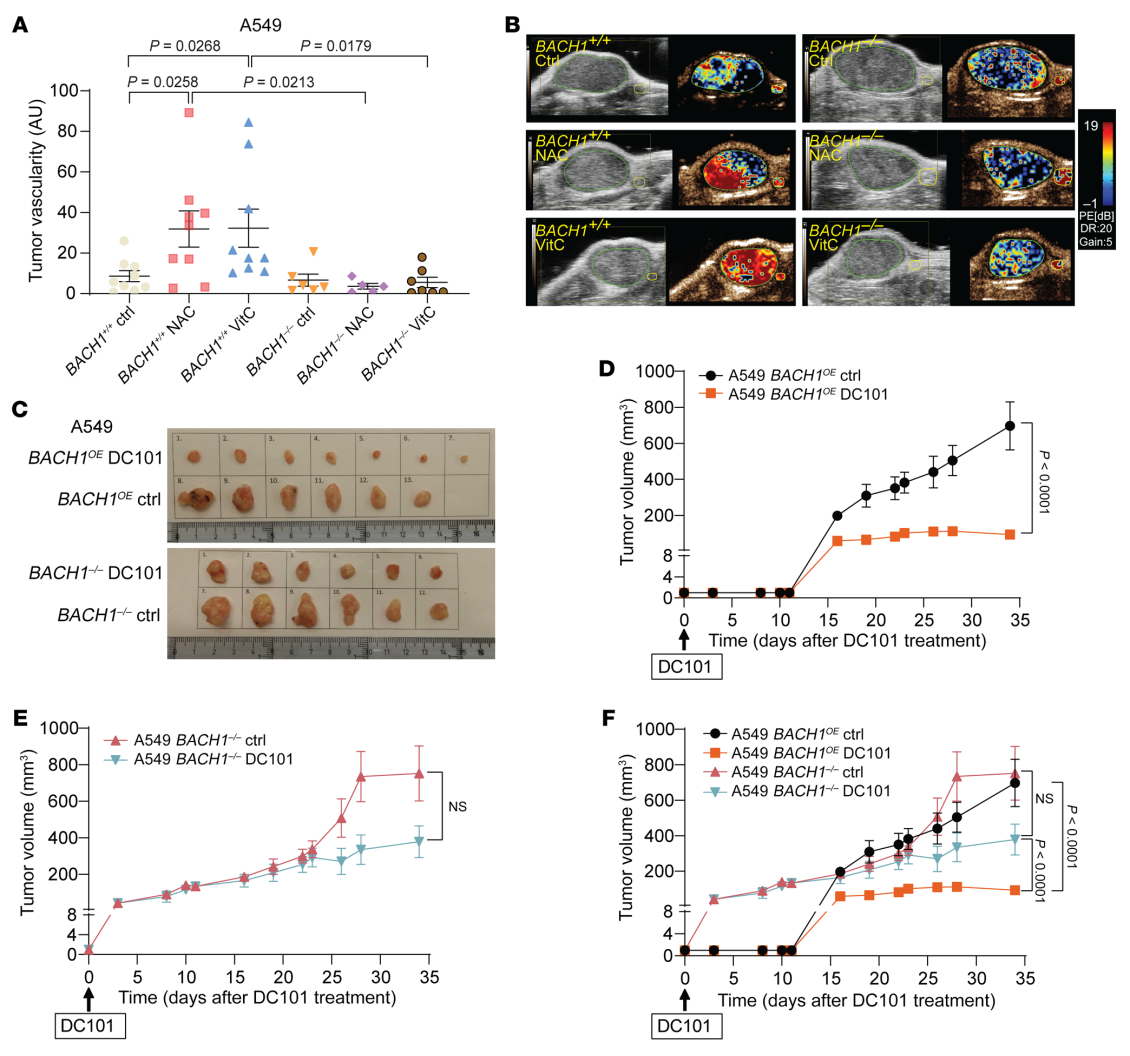
BACH1 increases tumor vascularity and the response to anti-VEGF therapy in xenografts. (**A**) Tumor vascularity (peak enhancement) in *NSG* mice injected s.c. with 5 × 10^5^ *BACH1^+/+^* or *BACH1^–/–^* A549 cells and administrated water (*n* = 9 and 6 for +/+ and –/–, respectively), 1 g/L NAC (*n* = 9 and 5), or 3.47 g/L VitC (*n* = 9 and 7) for 7 weeks. (**B**) Representative images of tumor vascularity from ultrasound imaging analyses. (**C**–**E**) Tumor growth in *NSG* mice injected s.c. with 5 × 10^5^ *BACH1*^OE^ (**C** and **D**) and *BACH1^–/–^* (**C** and **E**) A549 cells. When tumors were palpable, the mice were injected i.p. with PBS (*n* = 6 in **D** and **E**) and 40 mg/kg DC101 (**D**, *n* = 7; **E**, *n* = 6) 3 times per week for 5 weeks. Tumors were measured 3–5 times per week. (**F**) Curves from **D** and **E** are shown in the same graph. Data indicate the mean ± SEM. Statistical significance was determined by 1-way ANOVA with Tukey’s post hoc test for multiple comparisons (**A**) and 2-way ANOVA with Šidák’s post hoc test for multiple comparisons (**F**).

To determine whether antioxidant-mediated BACH1 activation is functionally involved in tumor angiogenesis, we administered NAC and VitC to *NSG* mice harboring *BACH1^+/+^* and *BACH1^–/–^* tumors and quantified tumor vascularity by ultrasound analysis. NAC and VitC administration increased tumor vascularity, and knockout of BACH1 abolished this effect (Figure 7, A and B, and Supplemental Figure 9C). VitE administration produced results that overlapped substantially with those of NAC and VitC, however, they were not statistically significant (Supplemental Figure 9D). Moreover, we argued that increased BACH1 expression might increase the response of tumors to antiangiogenic therapy. To test this possibility, we injected anti-VEGFR2 antibodies (DC101) into *NSG* mice harboring palpable *BACH1*^OE^ and *BACH1^–/–^* xenograft tumors. Following an initial growth, *BACH1*^OE^ tumors stopped growing in DC101-injected mice and continued to grow in saline-injected controls. The effect of DC101 on *BACH1^–/–^* tumors was not significant, although the drug tended to reduce a delayed tumor growth increase (Figure 7, C–F, and Supplemental Figure 9E). Reexpression of *BACH1* in *BACH1^–/–^* cells restored their sensitivity to DC101 (Supplemental Figure 9, F–H).

## Discussion

This study identifies BACH1 as an oxygen- and redox-sensitive transcription factor that controls tumor angiogenesis and vascularity and renders tumors sensitive to antiangiogenic therapy. Our data demonstrate that BACH1 in lung cancer cells was activated during hypoxia and in response to antioxidant administration through both transcriptional and posttranslational mechanisms. We show that BACH1 transcription was controlled directly by HIF1α (i.e., BACH1 is a transcriptional target of HIF1α) and that the posttranslational stabilization of BACH1 under hypoxia was HIF1α independent and likely mediated by reduced prolyl hydroxylation–dependent degradation, as BACH1 proteins accumulated substantially in response to prolyl hydroxylation inhibitors in both *HIF1A^+/+^* and *HIF1A^–/–^* cells, whereas BACH1 stabilization under reducing conditions — following antioxidant administration — was mediated by reduced heme-dependent degradation, as described in previous reports (14, 26, 27). Once at high levels, BACH1 acted directly as a transcription factor for a broad range of angiogenesis and glycolysis genes and could regulate these genes independently of HIF1α.

HIF1α gene and protein levels increased following antioxidant administration and was essential for antioxidant-induced increases in BACH1 gene and protein levels. We therefore propose that HIF1α- and BACH1-stimulated angiogenesis and glycolysis contribute to the ability of dietary, pharmacological, and endogenous NRF2-driven antioxidants to accelerate lung tumor progression and metastasis, as described earlier (13, 14, 28).

The finding that ROS-lowering doses of antioxidants increased HIF1α levels was surprising because VitC has been shown to reduce HIF1α levels and transcriptional targets in some cancer cells (21). The antioxidant-induced increase in HIF1α levels was also surprising because increased ROS production from mitochondria during hypoxia is known to increase HIF1α levels by inhibiting hydroxylation-dependent degradation (29, 30). A potential explanation for this discrepancy is that mitochondrial ROS production under hypoxia is a short-term response (hours) (31), whereas the current study analyzed effects after 7 days of antioxidant exposure. Moreover, *HIF1A* gene expression increased following antioxidant administration and probably contributed to the increased protein levels.

The finding that BACH1 stimulated lung tumor angiogenesis and correlated with angiogenesis gene and protein expression in human lung tumors raises the possibility that BACH1 could be a biomarker for predicting a better outcome from antiangiogenic therapy. Indeed, anti-VEGFR2 therapy stopped the growth of high-BACH1-expressing tumors but not that of low-BACH1-expressing tumors. Future studies should be able to address the efficacy of this approach in a clinical setting and could potentially extend beyond lung cancer, as we also observed correlations between BACH1 and angiogenesis gene expression in breast and kidney cancer.

## Methods

### Mice.

*Kras2^LSL/+^* mice were on a C57BL/6-129/Sv mixed genetic background (14); all controls were littermates. A low dose of *Cre*-adenovirus (5 × 10^5^ PFU, University of Iowa, Iowa City, Iowa, USA) were administered intranasally to 6–7-week-old male and female mice. For xenograft experiments, NOD-SCID-γ mice (NSG) (NOD.Cg-*Prkdc*^scid^*Il2rg*^tm1Wjl^/SzJ, from Charles River Laboratories) were transplanted s.c. with 5 × 10^5^ *BACH1^–/–^*, *BACH1*^OE^, or *BACH1^–/–^* *BACH1*^OE^ A549 cells. When tumors were detected (i.e., reached 1–3 mm in size), the mice were injected i.p. with DC101 (40 mg/kg, BE0060, Bio X Cell) 3 times per week; control mice were injected with PBS. Tumor volume was measured 3 or 5 times per week with an electronic caliper and calculated as width^2^ × length × 1/2, and tumors were weighed at the endpoint.

### Cell culture and reagents.

The following human cell lines were used: A549 (CRL-7909, ATCC); H838 (CRL-5844, ATCC); ZFN-generated *HIF1A*-knockout (*HIF1α^–/–^*) and control (*HIF1α^+/+^*) A549 cells (CLLS-1014, MilliporeSigma); CRISPR/Cas9-generated *BACH1*-knockout (*BACH1^–/–^*) and control (sg*dTomato*, *BACH1^+/+^*) A549 cells (14); and CRISPR/SAM-generated *BACH1*^OE^ and control (SAM-sg*Tom*, *BACH1*^WT^) A549 cells (14). Cell lines tested negative for mycoplasma and were cultured in DMEM low-glucose GlutaMax medium (21885-025, Life Technologies, Thermo Fisher Scientific) supplemented with 10% FBS (26140079, Thermo Fisher Scientific), 1% nonessential amino acids (NEAA) (11140-035, Life Technologies, Thermo Fisher Scientific), and 1% penicillin/streptomycin (15140122, Thermo Fisher Scientific) in either a normoxic (21% O_2_) or hypoxic (1% O_2_) cell incubator with 5% CO_2_ at 37°C for 7 days. The following reagents were used: NAC (A7250, MilliporeSigma), VitC (A5960, MilliporeSigma), Trolox (238813, MilliporeSigma), DMOG (D1070100MG, Frontier Scientific), and FG0041 (fibrogen, used previously in ref. 32).

### Lentivirus.

Cells were transduced with lentiviruses in the presence of polybrene (8 μg/mL, 107689-10G, MilliporeSigma) and selected with puromycin (1 μg/mL, A1113803, Thermo Fisher Scientific) for 48 hours or with blasticidin (7.5 μg/mL, A1113903, Thermo Fisher Scientific) for 7 days. Lentiviruses (pLV-EGFP-CMV-FLAG/gene) overexpressing *BACH1*, *HIF1A*, *EPAS1*, or *EGFP* (control) were produced by VectorBuilder.

### Mouse lung tumor organoids.

Mouse lung tumor tissue was dissected into approximately 1 mm^3^ fragments with sterile scissors and incubated at 37°C for 1 hour in Eppendorf tubes with 1 mL digestion medium (Advanced DMEM/F-12, 10% FBS, glutamine, HEPES, and penicillin/streptomycin (Thermo Fisher Scientific) supplemented with collagenase type IV (100 mg, MilliporeSigma) and dispase II (20 mg, Thermo Fisher Scientific). The cells were pelleted by centrifugation for 5 minutes at 200*g* and 4°C and resuspended in 150 μL Growth Factor Reduced Matrigel (356231, Corning). Domes of 50 μL cells/Matrigel suspension were pipetted into wells of a prewarmed 24-well plate and allowed to solidify for 10 minutes at 37°C. Prewarmed growth medium (same as the digestion medium without collagenase and dispase) supplemented with 10 μM RHO kinase inhibitor (Rocki, Y-27632, MilliporeSigma), insulin-transferrin-selenium supplement (MilliporeSigma), and TGF-βR inhibitor (A83-01, Thermo Fisher scientific) was added to the wells, and the plate was incubated at 37°C.

### Spheroid (3D) culture.

Cultured human cancer cells were trypsinized, counted, and mixed with Matrigel (356231, Corning). Domes of 25 μL cells/Matrigel suspension were pipetted into wells of a prewarmed 24-well plate and allowed to solidify for 10 minutes at 37°C. The cells were then cultured as described above for organoids.

### ROS measurements.

Cells were incubated with NAC and VitC for 7 days and seeded in white 96-well plates (5,000 cells/well). ROS were measured with the ROS-Glo-H2O2 assay (G8820, Promega). The ratio of GSH/GSSG was determined with the GSH/GSSG-Glo assay (V6611, Promega). Fluorescence was recorded with a Synergy multimode reader (BioTek).

### TCGA data analysis.

For analysis of data in TCGA, BACH1 expression data (RNA-Seq V2 RPKM-UQ) from 3,372 publicly available cases including 1,132 lung, 1,220 breast, and 1,020 kidney cancer cases were downloaded from the Genomic Data Commons (GDC) Application Programming Interface (API) using TCGAbiolinks R package (http://bioconductor.org/packages/TCGAbiolinks/). For each cancer type, cases were sorted into a high *BACH1* expression group (25% of the samples with the highest expression) and a low expression group (25% samples with the lowest expression). The expression of angiogenic genes was compared between the high *BACH1* and low *BACH1* expression groups using a 2-tailed Student’s *t* test. Angiogenic genes with a *P* value of less than 0.05 were retained, and their correlation with *BACH1* was calculated using Pearson’s correlation coefficient.

### Immunohistochemistry.

Human *KRAS*-mutant NSCLC biopsy sections and linked clinical data (Supplemental Table 2) for 20 patients were obtained from the Zhengzhou University Cancer Biobank. Samples were dehydrated, formalin-fixed, and paraffin-embedded, and 5 μm serial sections were mounted onto glass slides. Sections were incubated with primary antibodies recognizing BACH1 (sc-271211, Santa Cruz Biotechnology, 1:200); VEGFA (sc-7269, Santa Cruz Biotechnology, 1:200); and VEGFR2 (sc-6251, Santa Cruz Biotechnology, 1:200) at 4°C overnight, followed by incubation with HRP-conjugated secondary antibodies (Zhong-shan Golden Bridge) for 1 hour. Next, the sections were stained with 3,3′-diaminobenzidine and hematoxylin. The stained sections were then scanned using a Panoramic Confocal microscope (3DHistech). Quantification of BACH1, VEGFA, and VEGFR2 staining intensity was performed using Aipathwell digital pathology AI-based image analysis software. Each sample was assigned a score on the basis of modified H-scores [H-scores =∑ (pi × i) = (percentage of weak intensity × 1) + (percentage of moderate intensity × 2) + (percentage of strong intensity × 3)] (33–36).

### CUT&Tag chromatin profiling.

CUT&Tag was used to assess the genome-wide chromatin enrichment of BACH1 and H3K27ac in A549 3D spheroids. CUT&Tag was performed on 10^5^ cells from 3D spheroid cultures essentially as described previously (37) using digitonin (MilliporeSigma, D5628) for cell permeabilization and concanavalin A–coated magnetic beads (Bangs Laboratories, BP531) for immobilization. Two biological replicates were used for all experiments. The primary antibodies used were H3K27ac (Abcam, ab4729); BACH1 (R&D Systems, AF5776); and HIF1α (Novus Biologicals, NB100-134). The secondary antibodies used were anti-goat (MilliporeSigma, SAB3700280) and anti-rabbit (EpiCypher, 13-0047). Samples were incubated with pAG-Tn5 (EpiCypher, 15-1117) for 1 hour. After tagmentation, the cleaved DNA was extracted using the DNA Clean & Concentrator-5 Kit (Zymo Research, D4013). IDT primers (Illumina, 20027213) and PCR enzyme mix (New England BioLabs [NEB], M0541S) were used for library preparation, and AMPure beads (Beckman Coulter, A63881) were used for PCR cleanup. DNA concentration was measured by Qubit (Invitrogen, Thermo Fisher Scientific, Q32851). Library samples were sequenced on the NextSeq 2000 (PE100) platform (BEA, Karolinska Institutet) using pair-ended output.

### CUT&Tag sequencing data analysis.

Sequencing files (FASTQ) were aligned to the GRCh37/hg19 human reference genome using Bowtie2 (38). Peak calling was computed via MACS2 (39). The sequencing tags (SAM) and peak file (BED) were imported into HOMER for statistical analysis (40). BedGraph files were imported into IGV software for data visualization. A total of 10^7^ tags were used as a normalization factor to compare treatments and groups. Motif analysis was done with HOMER (findMotifs.pl). Peak coverage was calculated with the HOMER tool Histograms Tag (annotatePeaks.pl) and visualized in R. The BACH1 peak distribution was based on the HOMER annotation file. Changes in individual peak tags (percentage) were calculated from normalized HOMER output data. The HIF1α genome-wide heatmap was calculated via Deeptools (41).

### Reverse transcription quantitative PCR.

Cell recovery solution (DLW354253, MilliporeSigma) was used to isolate cells from Matrigel. RNA was isolated with the RNeasy Plus Mini kit (74136, QIAGEN), and cDNA was synthesized using the iScript cDNA synthesis kit (170-889, Bio-Rad). Gene expression was analyzed with SYBR Green Master Mix (KCQS00, MilliporeSigma) on a CFX384 Real-Time System (Bio-Rad) using the following predesigned KiCqStart SYBR-Green Primers (all from MilliporeSigma): *BACH1* (H_*BACH1*_1), *HIF1A* (H_*HIF1A*_1), *HIF2A* (H_*EPAS1*_1), *VEGFA* (H_*VEGFA*_1), *VEGFB* (H_*VEGFB*_1), *VEGFC* (H_*VEGFC*_1), *VEGFD* (H_*FIGF*_1), *VEGFR1* (H_*FLT1*_1), *VEGFR2* (H_*KDR*_1), *VEGFR3* (H_*FLT4*_1), *NRP1* (H_*NRP1*_1), *NRP2* (H_*NRP2*_1), *GLUT1* (H_*SLC2A1*_1), *GLUT3* (H_*SLC2A3*_1), *HK1* (H_*HK1*_1), *HK2* (H_*HK2*_1), *PGK1* (H_*PGK1*_1), *PDK1* (H_*PDK1*_1), *PFKP* (H_*PFKP*_1), *PFKFB2* (H_*PFKFB2*_1), *PFKFB3* (H_*PFKFB3*_1), *PKM2* (H_*PKM2*_1), *PKLR* (H_*PKLR*_1), *LDHA* (H_*LDHA*_1), *FGF2* (H_*FGF2*_1), *FGF7* (H_*FGF7*_1), *FGF9* (H_*FGF9*_1), *FGFR1* (H_*FGFR1*_1), *FGFR2* (H_*FGFR2*_1), *FGFR3* (H_*FGFR3*_1), *FGFR4* (H_*FGFR4*_1), *FGFR1OP* (H_*FGFR1OP*_1), *EGF* (H_*EGF*_1), *EGFR* (H_*EGFR*_1), *EGFL7* (H_*EGFL7*_1), *EFNA5* (H_*EFNA5*_1), *ANGPT1* (H_*ANGPT1*_1), *ANGPT2* (H_*ANGPT2*_1), *ANGPTL1* (H_*ANGPTL1*_1), *ANGPTL2* (H_*ANGPTL2*_1), *ANGPTL4* (H_*ANGPTL4*_1), *Bach1* (M_*Bach*_1), *Vegfa* (M_*Vegfa*_1), *Vegfb* (M_*Vegfb*_1), *Vegfd* (M_*Figf*_1), *Vegfr1* (M_*Flt1*_1), *Vegfr2* (M_*Kdr*_1), *Vegfr3* (M_*Flt4*_1), *Nrp1* (M_*Nrp1*_1), *Nrp2* (M_*Nrp2*_1), *Hk1* (M_*Hk1*_1), *Hk2* (M_*Hk2*_1), *Pgk1* (M_*Pgk1*_1), *Pdk1* (M_*Pdk1*_1), *Pfkp* (M_*Pfkp*_1), *Pfkfb2* (M_*Pfkfb2*_1), *Pfkfb3* (M_*Pfkfb3*_1), *Pkm2* (M_*Pkm2*_1), *Pklr* (M_*Pklr*_1); *ACTB* (H_*ACTB*_1), *Actb* (M_*Actb*_1) was the reference gene.

### Western blotting.

Cell recovery solution (DLW354253, MilliporeSigma) was used to isolate cells from Matrigel. Cells were lysed in Laemmli buffer supplemented with β-mercaptoethanol. Equal amounts of proteins were resolved on 4%–20% or 10% Mini-PROTEAN TGX Stain-Free gels (456–8036, Bio-Rad) and electrotransferred onto nitrocellulose membranes (0.2 μm, 1704158, Bio-Rad). The membranes were blocked with 5% milk in TBST and incubated with primary antibodies overnight and secondary antibodies for 1 hour at room temperature. The primary antibodies used were: BACH1 (sc-271211, Santa Cruz Biotechnology, 1:1,000); VEGFR2 (sc-6251, Santa Cruz Biotechnology, 1:200); NRP2 (sc-13117, Santa Cruz Biotechnology, 1:200); actin (MilliporeSigma, A2228, 1:1,000); H3K27ac (ab4729, Abcam, 1:2,000); HIF1α (14179, 3716, Cell Signaling Technology, 1:1,000); and HIF2α (PA1-16510, Invitrogen, Thermo Fisher Scientific, 1:1,000). The secondary antibodies used were: rhodamine (TRITC) AffiniPure goat anti–mouse IgG (H+L) (115-025-003, 1:10,000) and peroxidase AffiniPure goat anti–rabbit IgG (H+L) (111-035-003, 1:10 000) from Jackson ImmunoResearch Laboratories. Western ECL substrate (1705061, Bio-Rad) was used for protein band detection with the ChemiDoc Touch Imaging System (1708370, Bio-Rad). Band densities were quantified with Image Lab Software.

### High-frequency ultrasound imaging.

*NSG* mice were s.c. transplanted with 5 × 10^5^ *BACH1^–/–^* and *BACH1^+/+^* A549 cells and received NAC (1 g/L) or VitC (3.47 g/L) in the drinking water or VitE (DL-α-tocopheryl acetate) in the chow (Lantmännen) at a dose of 0.5 g/kg chow (61.5 mg/kg body weight), calculated from an observed daily food intake (28). Ultrasound imaging of tumors was performed on a Vevo LAZR-X Imaging Station (VisualSonics) using a high-frequency ultrasound probe MX250 (15–30 MHz, 75 μm image axial resolution). Mice were anesthetized with 1.5% isoflurane and medical air flow of 2 L/minutes during the imaging process; hair over the imaged area was removed using a depilatory cream; and ultrasound gel (Parker Laboratories) was applied over the region of interest. Tumor size quantification was performed using 18 MHz B-mode. 3D images were acquired with a 3D acquisition motor scanned along the vertical axis. 3D volumetric quantification was performed to integrate multiple 2D ultrasound images. Nonlinear contrast imaging was acquired at 18 MHz frequency, 10% power, 30 dB contrast gain, and a 20/s frame rate — immediately after an i.v. bolus injection of 50 μL nontargeting microbubbles (2 × 10^9^/mL, VevoMicroMarker Contrast Agent, VisualSonics). Tumor perfusion/vascularity (peak enhancement) was quantified with VevoCQ Software (VisualSonics).

### Data availability.

Sequence data from the CUT&Tag experiments have been deposited in the NCBI’s Gene Expression Omnibus (GEO) database (GEO GSE209958). Values for all data points in graphs can be found in the Supplemental Supporting Data Values file.

### Statistics.

Data are shown as the mean ± SEM. GraphPad Prism version 8 (GraphPad Software) was used for statistical analyses. Statistical significance was determined using 2-way ANOVA for tumor volume, an unpaired, 2-tailed Student’s *t* test when comparing only 2 groups, and 1-way ANOVA followed by Tukey’s post hoc test for all other comparisons. The relationship between BACH1 and VEGFA and VEGFR2 expression was analyzed using the Pearson’s correlation test. A *P* value of less than 0.05 was considered a significant difference. Experiments were repeated 2–4 times unless stated otherwise; *n* values indicate the number of biological replicates.

### Study approval.

Animal experiments were approved by the research animal ethics committees in Gothenburg and Linköping, Sweden. Analyses of human tissues were approved by the ethics committee of The First Affiliated Hospital of Zhengzhou University (approval ZBMT001), and human experiments were performed in accordance with Helsinki Declaration principles. Human tissues were deidentified, and all patients provided written informed consent for the use of their biopsy samples.

## Author contributions

TW designed the study, performed experiments, interpreted data, created figures, and wrote the manuscript. YQD and GQZ performed and analyzed human NSCLC experiments. ZH, NL, and E Treuter designed, performed, and analyzed CUT&Tag experiments. HDY provided advice and performed mouse experiments. E Tüksammel, HC, XJY, and S Schmidt performed mouse experiments. CTJ designed experiments. YZ performed and analyzed photoacoustic imaging experiments. XQ, JJH, and S Strömblad provided technical support.XX and S Schilsio provided advice and reagents. HQ supervised and provided infrastructure and advice. MOB conceived the study, interpreted data, supervised the study, provided funding, and wrote the manuscript. All authors read and commented on the manuscript.

## Supplementary Material

Supplemental data

Supporting data values

## Version 1 08/31/2023

In-Press Preview

## Version 2 10/16/2023

Electronic publication
