# Supplementary material for: Antioxidants stimulate BACH1-dependent tumor angiogenesis
Source: J Clin Invest. 2023 Oct 16;133(20):e169671. doi: 10.1172/JCI169671 (PMC10575724; doi:10.1172/JCI169671)
Supplement: Supplemental data [file jci-133-169671-s104.pdf]

Supplemental material

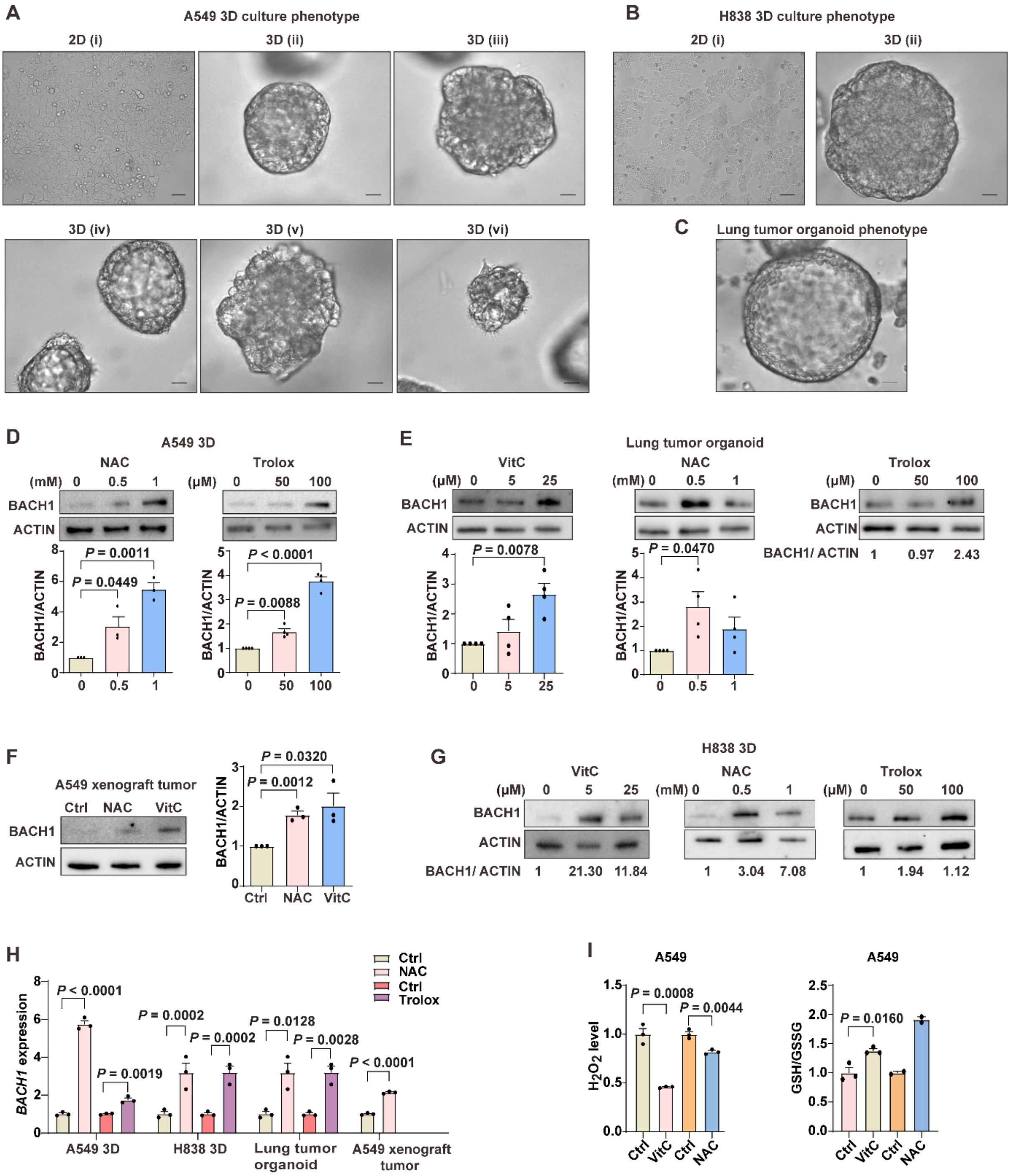

Supplemental Figure 1. NAC, VitC, and Trolox increase BACH1 gene and protein levels in human lung cancer cell spheroids and mouse tumor-derived organoids. (**A**) Bright-field images showing morphology of A549 cells cultured in 2D (i) and 3D spheroids (ii-vi). Scale bar, 100 μm. (**B**) Bright-field images showing morphology of H838 cells cultured in 2D (i) and 3D spheroids (ii). (**C**) Image showing the typical morphology of a mouse lung tumor organoid. (**D**) BACH1 protein levels in A549 spheroids incubated with NAC (left) and Trolox (right) for 7 days (n = 3-4 experiments). (**E**) BACH1 protein levels in mouse lung tumor organoids incubated 7 days with VitC,

NAC, and Trolox (n = 4 experiments) for NAC and VitC; BACH1/ACTIN ratio of the experiment with Trolox is indicated. (**F**) BACH1 protein levels in A549 xenograft tumors isolated from mice following administration of 1 g/l NAC and 3.47 g/l VitC in the drinking water for 7 weeks (n = 3 mice/3 experiments). (**G**) BACH1 protein levels in H838 spheroids incubated with 25 µM VitC, 1 mM NAC, and 100 µM Trolox for 7 days. The BACH1/ACTIN ratio is indicated. (**H**) RT-qPCR of *BACH1* in A549 and H838 spheroids, and mouse lung tumor organoids incubated 7 days with 1 mM NAC and 100 µM Trolox, and A549 xenograft tumors from mice administrated NAC (1 g/l) in the drinking water for 7 weeks (n = 3 experiments). (**I**) H_2O_2 and GSH/GSSG levels in A549 cells incubated 7 days with 1 mM NAC and 25 µM VitC (n = 2-3 experiments). Scale bars, 100 µm. Data are mean and SEM. Statistics: Two-tailed unpaired Student's *t* test (panel H, I) and one-way ANOVA with Tukey's post-hoc test for multiple comparisons (panel D, E, F).

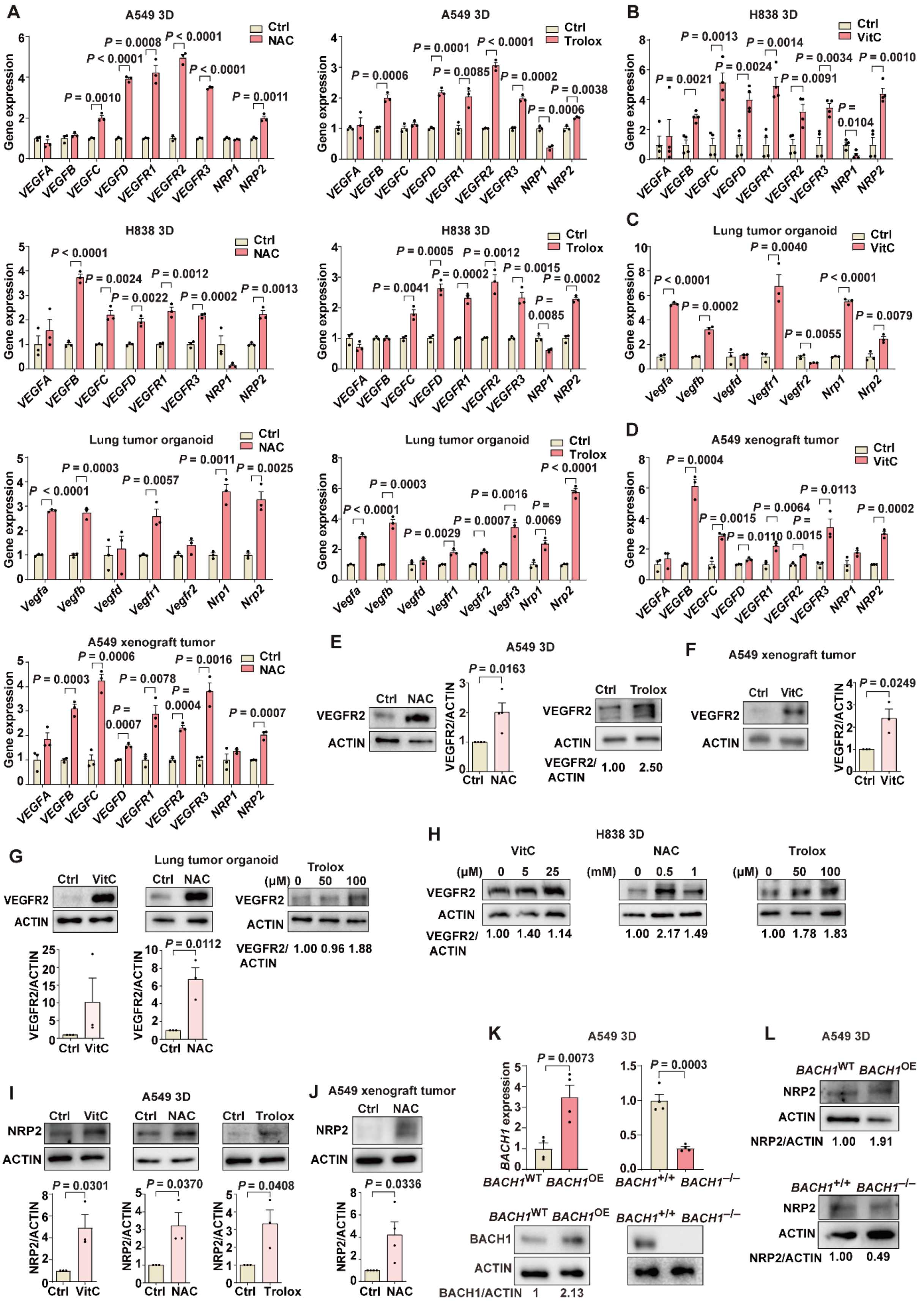

Supplemental Figure 2. BACH1 controls expression of angiogenesis genes in lung tumor organoids and spheroids under normoxic conditions. (**A**) RT-qPCR

of angiogenesis genes in A549 spheroids incubated 7 days with 1 mM NAC (left) and 100 μM Trolox (right) (n = 3 experiments). (**B and C**) RT-qPCR of angiogenesis genes in H838 spheroids (B) and lung tumor organoids (C) incubated with 25 μM VitC, 1 mM NAC, and 100 μM Trolox (n = 2 experiments). (**D**) RT-qPCR of angiogenesis genes in A549 xenograft tumors isolated from mice administered 3.47 g/l VitC or 1 g/l NAC in the drinking water for 7 weeks (n = 3 experiments). (**E**) Left, VEGFR2 protein levels in spheroids incubated with 1 mM NAC (n = 4 experiments). Right, VEGFR2 protein levels in spheroids incubated with 100 μM Trolox. (**F**) Left, VEGFR2 protein levels in xenograft tumors isolated from mice administered 3.47 g/l VitC in the drinking water for 7 weeks. Right, VEGFR2 levels by densitometry (n = 3 mice/3 experiments). (**G**) Top, VEGFR2 protein levels in mouse lung tumor organoids incubated with 25 μM VitC, 1 mM NAC, and 50 and 100 μM Trolox. Bottom, BACH1 levels by densitometry in three VitC and NAC experiments; for Trolox VEGFR2/ACTIN ratio is indicated. (**H**) VEGFR2 protein levels in H838 spheroids incubated with antioxidants for 7 days. (**I**) NRP2 protein levels in spheroids incubated with 25 μM VitC, 1 mM NAC, and 100 μM Trolox (n = 3 experiments). (**J**) NRP2 protein levels in xenograft tumors isolated from mice administered 1 g/l NAC in the drinking water for 7 weeks (n = 4 mice; 3 experiments). (K) Left top, RT-qPCR of *BACH1* in *BACH1*^{OE} and control cells with normal BACH1 expression (*BACH1*^{WT}). Bottom, BACH1 protein levels. Right top, RT-qPCR of *BACH1* in *BACH1*$^{+/+}$ and *BACH1*$^{-/-}$ spheroids. Bottom, BACH1 protein levels. (**L**) NRP2 protein levels in *BACH1*^{WT} and *BACH1*^{OE} (left) and *BACH1*$^{+/+}$ and *BACH1*$^{-/-}$ (right) spheroids. Data are mean and SEM. Statistics: Two-tailed unpaired Student's *t* test (panel A, B, C, D, E, F, G, I, J, K).

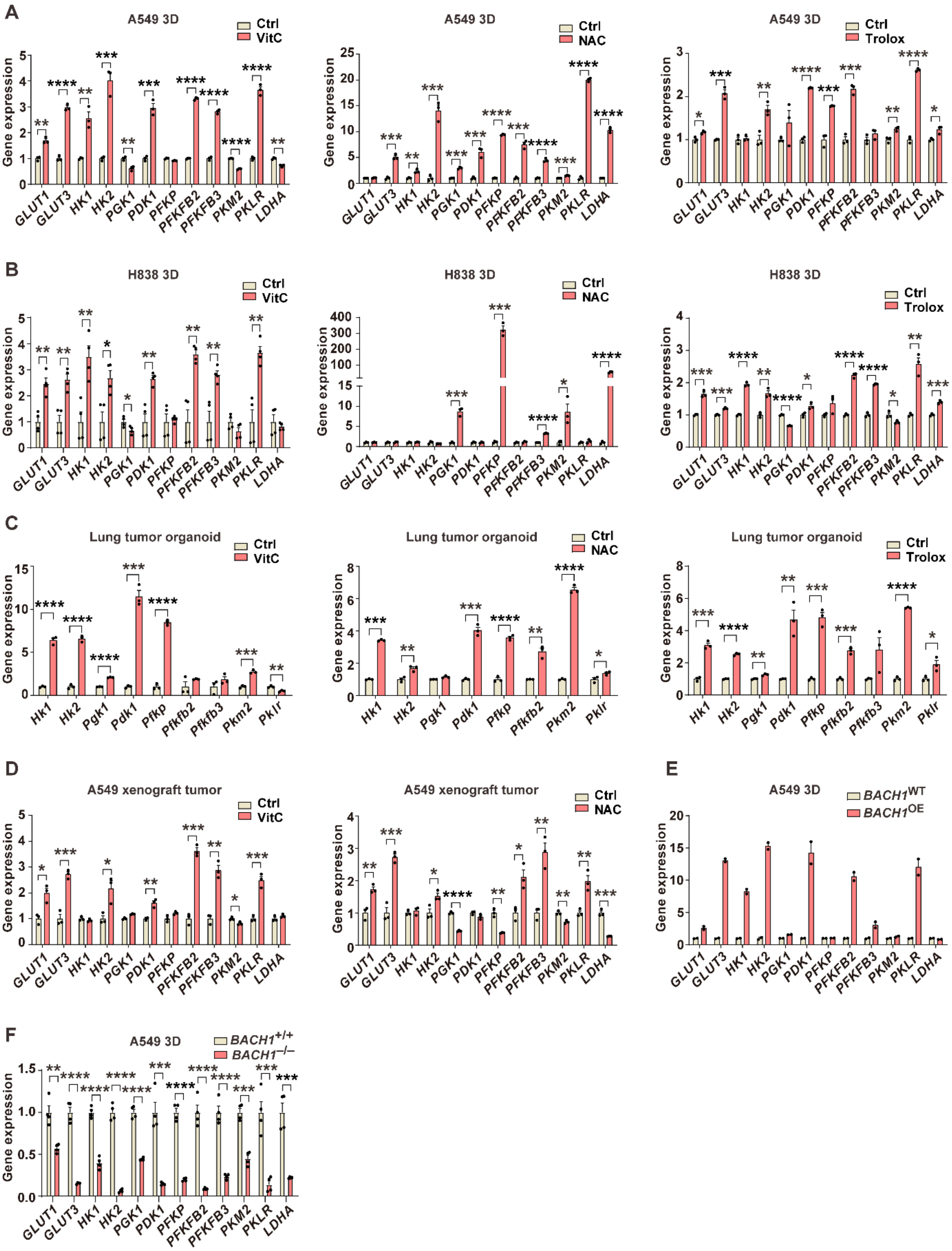

Supplemental Figure 3. BACH1 controls expression of a broad range of glycolysis genes in spheroids, organoids, and xenografts. (**A–C**) RT-qPCR analyses of glycolysis-related genes in A549 spheroids (A), H838 spheroids (B), and mouse lung tumor organoids. (C) incubated 7 days with 25 µM VitC (left), 1 mM NAC (middle), and 100 µM Trolox (right) (n = 2-3 experiments). (**D**) RT-qPCR of glycolysis-related genes in A549 xenograft tumors isolated from mice administered 3.47 g/l VitC or 1 g/l NAC in the drinking water for 7 weeks (n = 3 experiments). (**E and F**) RT-qPCR

of glycolysis-related genes in spheroids generated from *BACH1*^{WT} and *BACH1*^{OE} A549 cells (E) (n = 2 experiments); and from *BACH1*$^{+/+}$ and *BACH1*$^{-/-}$ A549 cells (F) (n = 4 experiments). Data are mean and SEM. Statistics: Two-tailed unpaired Student's *t* test (panel A, B, C, D, F). * P < 0.05; ** P < 0.01; *** P < 0.005; **** P < 0.001.

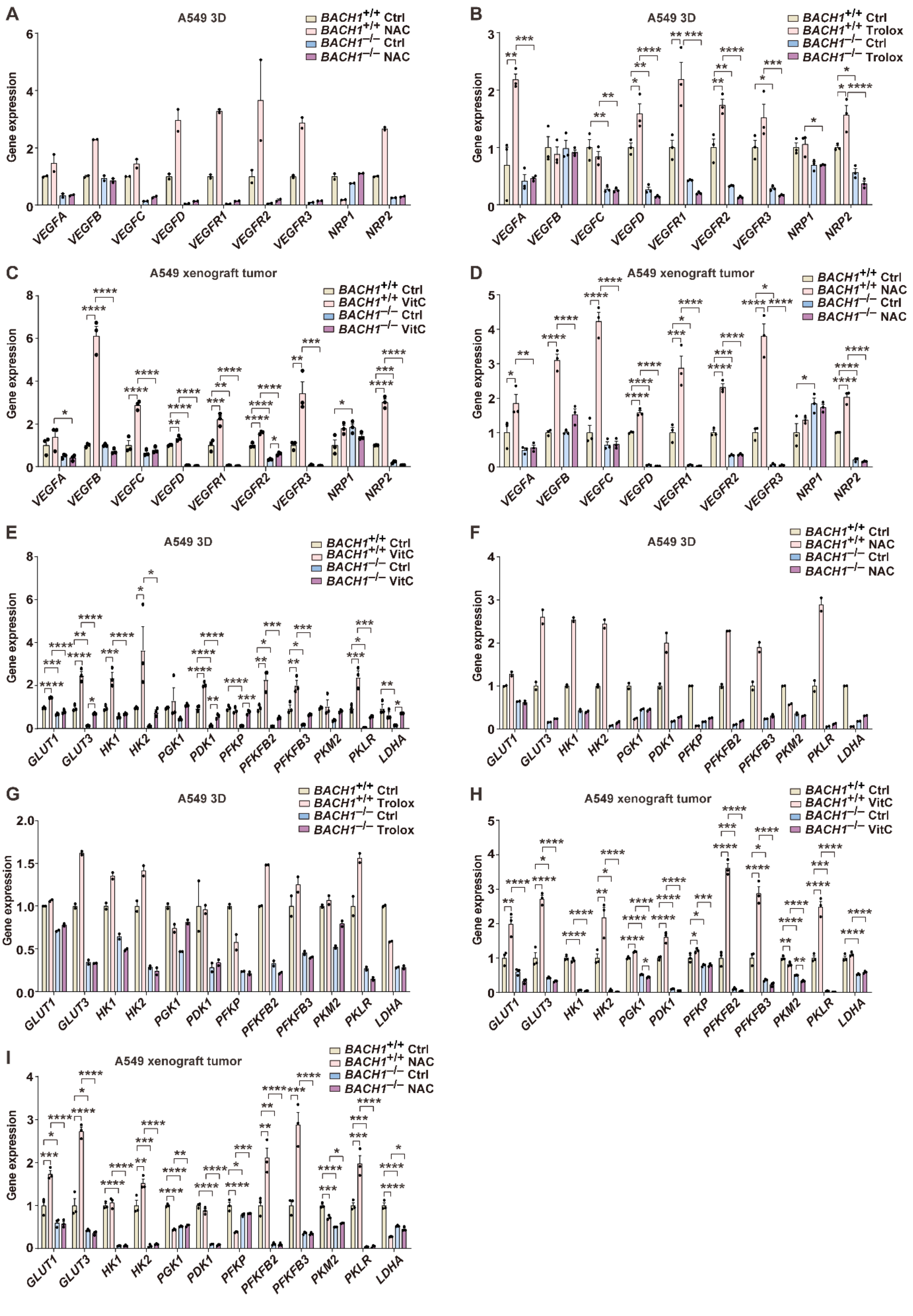

Supplemental Figure 4. BACH1 controls basal and antioxidant-induced expression of angiogenesis and glycolysis genes in lung cancer cell spheroids

and xenograft tumors. (**A–D**) RT-qPCR analyses of angiogenesis-related genes in *BACH1*$^{+/+}$ and *BACH1*$^{-/-}$ A549 spheroids incubated with 1 mM NAC (A) and 100 µM Trolox (B); and *BACH1*$^{+/+}$ and *BACH1*$^{-/-}$ A549 xenograft tumors isolated from mice following administration of 3.47 g/l VitC (C) and 1 g/l NAC (D) for 7 weeks (n = 2-3 experiments). (**E–I**) RT-qPCR analyses of glycolysis-related genes in *BACH1*$^{+/+}$ and *BACH1*$^{-/-}$ A549 spheroids incubated with 25 µM VitC (E), 1 mM NAC (F), and 100 µM Trolox (G) for 7 days; and *BACH1*$^{+/+}$ and *BACH1*$^{-/-}$ A549 xenograft tumors isolated from mice following administration of 3.47 g/l VitC (H) and 1 g/l NAC (I) for 7 weeks (n = 2-3 experiments). Data are mean and SEM. Statistics: One-way ANOVA with Tukey's post-hoc test for multiple comparisons (panel B, C, D, E, H, I). * P < 0.05; ** P < 0.01; *** P < 0.005; **** P < 0.001.

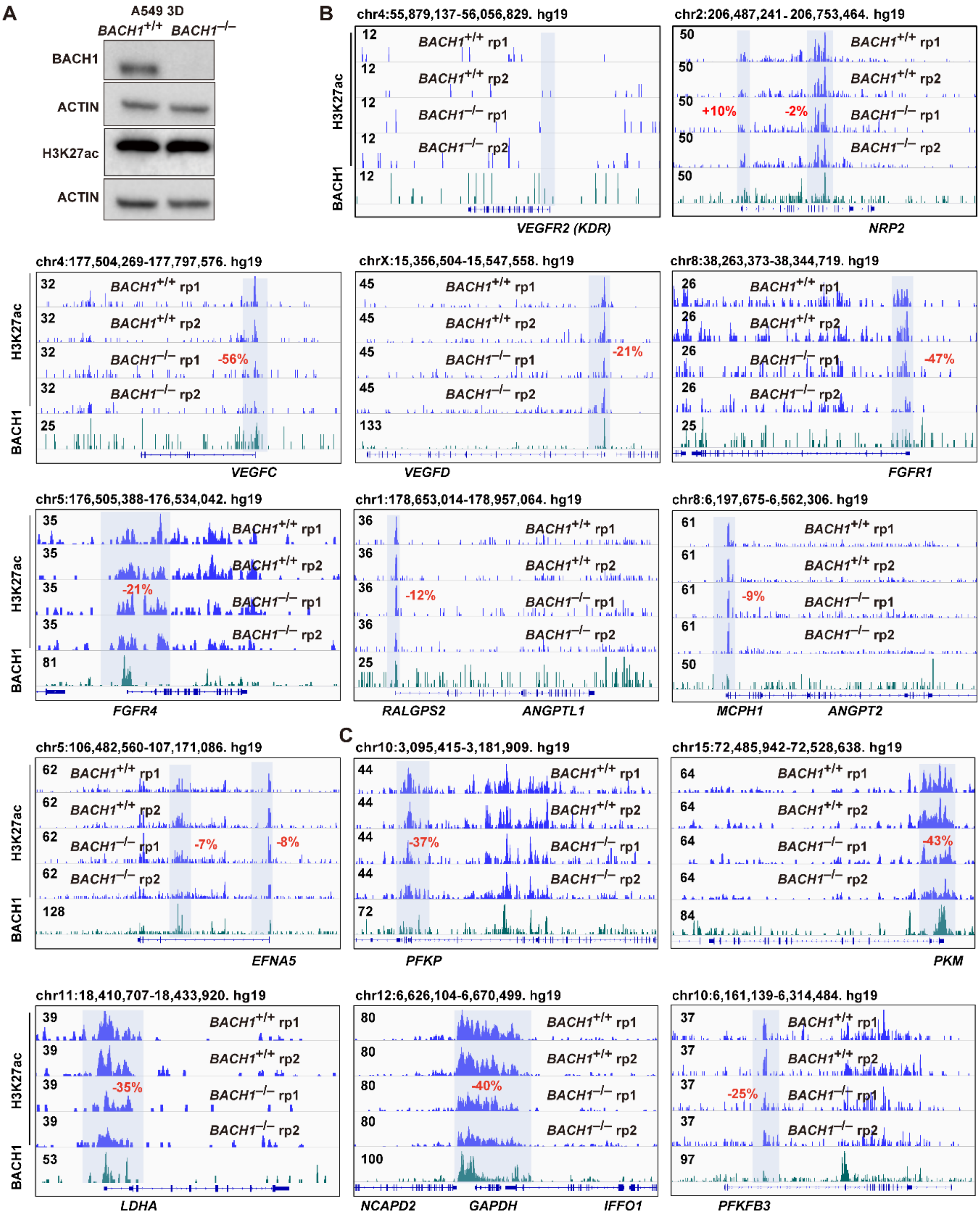

Supplemental Figure 5. BACH1-mediated expression of angiogenesis and glycolysis genes correlates with BACH1-dependent epigenetic changes at promoter regions. (**A**) Western blot analysis showing basal protein amounts of BACH1 and H3K27ac in *BACH1*$^{+/+}$ and *BACH1*$^{-/-}$ A549 spheroids. (**B and C**) Integrative Genomics Viewer (IGV) tracks showing H3K27ac levels at the indicated angiogenesis (B, 9 panels) and glycolysis (C, 5 panels) gene loci in *BACH1*$^{+/+}$ and *BACH1*$^{-/-}$ A549 spheroids. BACH1 peaks are shown at the bottom to indicate overlap with H3K27ac-marked regions. Regions with significant H3K27ac changes in *BACH1*$^{-}$

$^{/-}$ compared with *BACH1*$^{+/+}$ A549 spheroids are highlighted in blue; the percent change is indicated with red numbers.

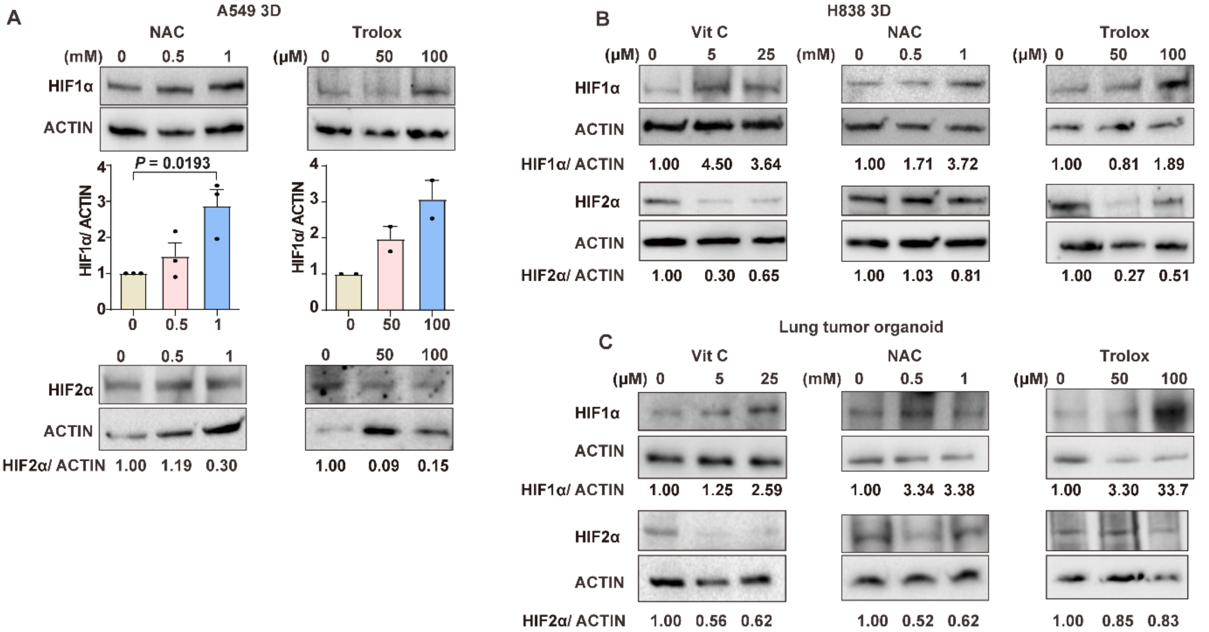

Supplemental Figure 6. The antioxidants VitC, NAC, and Trolox increase HIF1α but not HIF2α expression under normoxia in A549 and H838 spheroids and in organoids from mouse KRAS-induced lung tumors. (**A**) Western blots showing levels of HIF1α (top) and HIF2α (bottom) expression in A549 spheroid incubated with NAC (left) and Trolox (right) for 7 days. Middle, amounts of HIF1α determined by densitometry of protein bands (n = 2-3 experiments); HIF2α/ACTIN densitometry ratios are indicated. (**B and C**) Western blots showing levels of HIF1α (top) and HIF2α (bottom) in H838 spheroids (B) and mouse lung tumor organoids (C) incubated with VitC (left), NAC (middle), and Trolox (right) for 7 days. HIF1α/ACTIN and HIF2α/ACTIN densitometry ratios are indicated. ACTIN was the loading control. Data are mean and SEM. Statistics: One-way ANOVA with Tukey's post-hoc test for multiple comparisons (panel A).

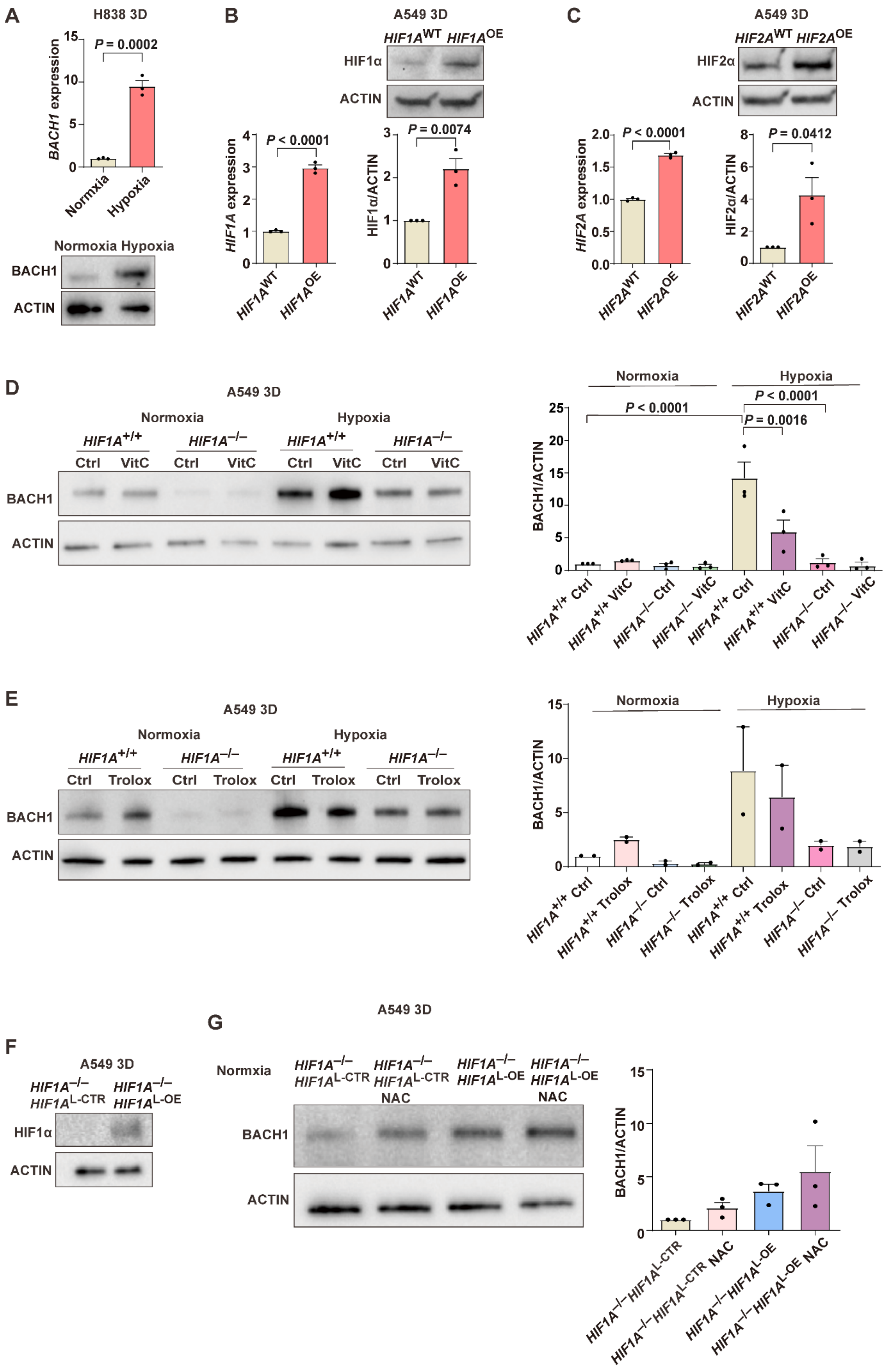
A
H838 3D
P = 0.0002
BACH1 expression
Normxia
Hypoxia
Normoxia Hypoxia
BACH1
ACTIN
B
A549 3D
HIF1A^WT HIF1A^OE
HIF1α
ACTIN
P < 0.0001
HIF1A expression
P = 0.0074
HIF1α/ACTIN
C
A549 3D
HIF2A^WT HIF2A^OE
HIF2α
ACTIN
P < 0.0001
HIF2A expression
P = 0.0412
HIF2α/ACTIN
D
A549 3D
Normoxia
Hypoxia
HIF1A+/+
HIF1A−/−
Ctrl
VitC
BACH1
ACTIN
P < 0.0001
P = 0.0016
BACH1/ACTIN
E
A549 3D
Trolox
BACH1/ACTIN
F
A549 3D
HIF1A−/− HIF1A^L-CTR
HIF1A−/− HIF1A^L-OE
HIF1α
ACTIN
G
A549 3D
Normxia
HIF1A−/− HIF1A^L-CTR NAC
HIF1A−/− HIF1A^L-OE NAC
BACH1
ACTIN
BACH1/ACTIN

Supplemental Figure 7. BACH1 expression under both normoxia and hypoxia is HIF1α dependent. (**A**) RT-qPCR (top) and western blot (bottom) showing BACH1 gene and protein expression in H838 spheroids incubated for 7 days under normoxia (21% O_2) and hypoxia (1% O_2) (n = 2 experiments). (**B**) Control experiment documenting increased HIF1α expression by RT-qPCR (left) and western blot (right, top) in $HIF1A^{WT}$ and $HIF1A^{OE}$ A549 spheroids. Bottom right, amounts of HIF1α determined by densitometry of protein bands (n = 3 experiments). (**C**) Analyses of $HIF2A^{WT}$ and $HIF2A^{OE}$ A549 spheroids in similar experiments as in panel B. (**D and E**) Left, western blot showing BACH1 expression in $HIF1A^{-/-}$ and control $HIF1A^{+/+}$ A549 spheroids incubated for 7 days with 25 mM VitC (D), 100 µM Trolox (E), and control medium (D and E) under normoxia (21% O_2) and hypoxia (1% O_2). Right, amounts of BACH1 determined by densitometry of protein bands (n = 2-3 experiments). (**F**) Western blot documenting restored HIF1α expression in $HIF1A^{-/-}$ A549 spheroids upon lentiviral transduction with a cDNA encoding human HIF1a ($HIF1A^{L\text{-}OE}$); control cells were transduced with EGFP ($HIF1A^{L\text{-}CTR}$). (**G**) Left, western blot showing BACH1 expression in $HIF1A^{-/-}HIF1A^{L\text{-}OE}$ and control $HIF1A^{-/-}HIF1A^{L\text{-}CTR}$ A549 spheroids incubated with 1 mM NAC or control medium for 7 days. Right, amounts of BACH1 determined by densitometry of protein bands (n = 3 experiments). ACTIN was the loading control in western blots. ns, not significant. Data are mean and SEM. Statistics: Two-tailed unpaired Student's *t* test (panel A, B, C) and one-way ANOVA with Tukey's post-hoc test for multiple comparisons (panel D).

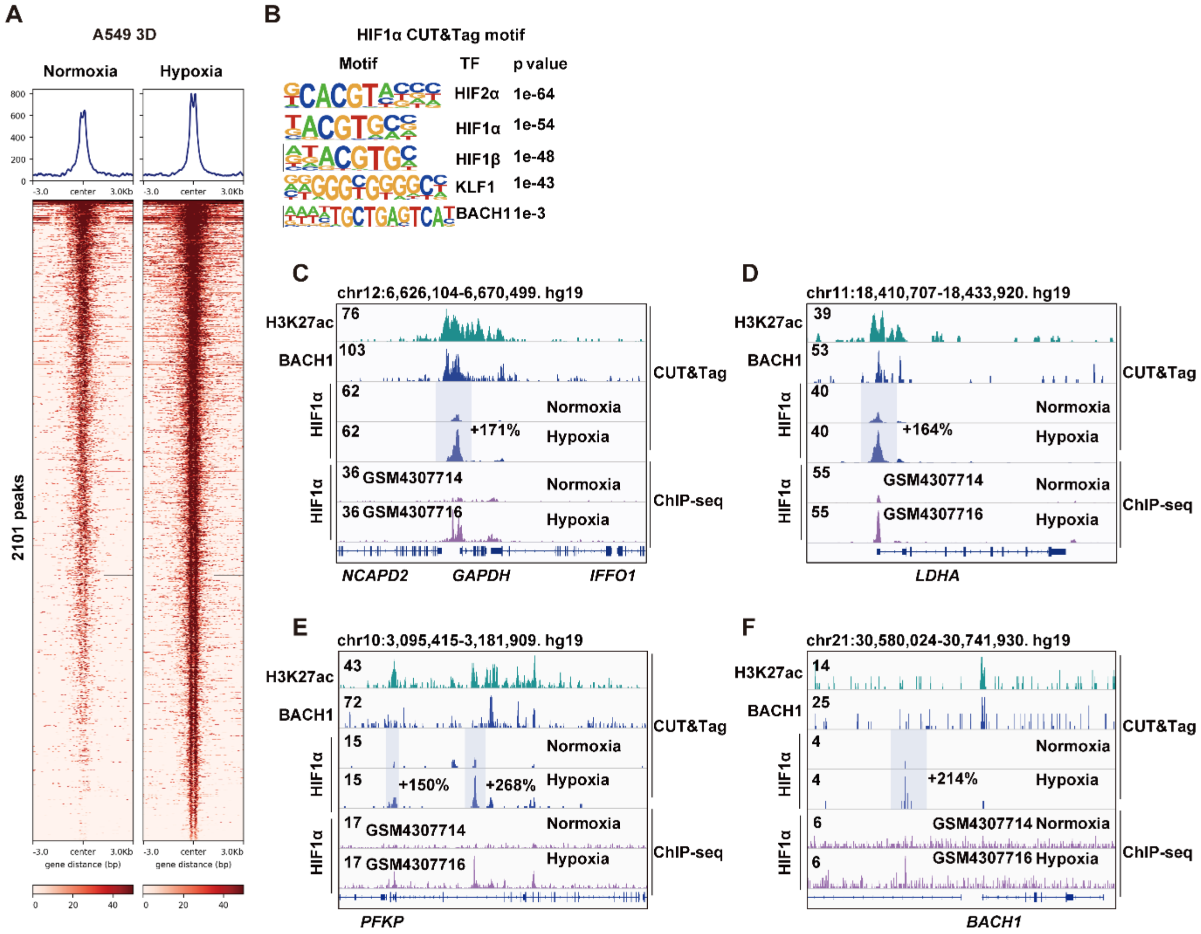

Supplemental Figure 8. BACH1 expression under both normoxia and hypoxia is HIF1α dependent. Related to Figure 3. (**A**) Genome-wide heatmap of HIF1α chromatin enrichment under normoxia (21% O_2) and hypoxia (1% O_2) for 7 days in A549 spheroids. (**B**) Transcription factor DNA-binding motif analysis of HIF1α CUT&Tag peaks. (**C–F**) IGV genome browser tracks showing HIF1α occupancy and levels at the indicated glycolysis-related gene loci (C–E) and at the BACH1 locus (F) in A549 spheroids cultured under normoxia (21% O_2) and hypoxia (1% O_2) for 7 days. H3K27ac and BACH1 tracks are shown at the top to indicate the genome environment. Previously published HIF1α ChIP-seq data are indicated at the bottom to compare with the CUT&Tag data. Regions with significant changes in HIF1α occupancy in response to hypoxia are highlighted in blue; the percent change is indicated with black numbers.

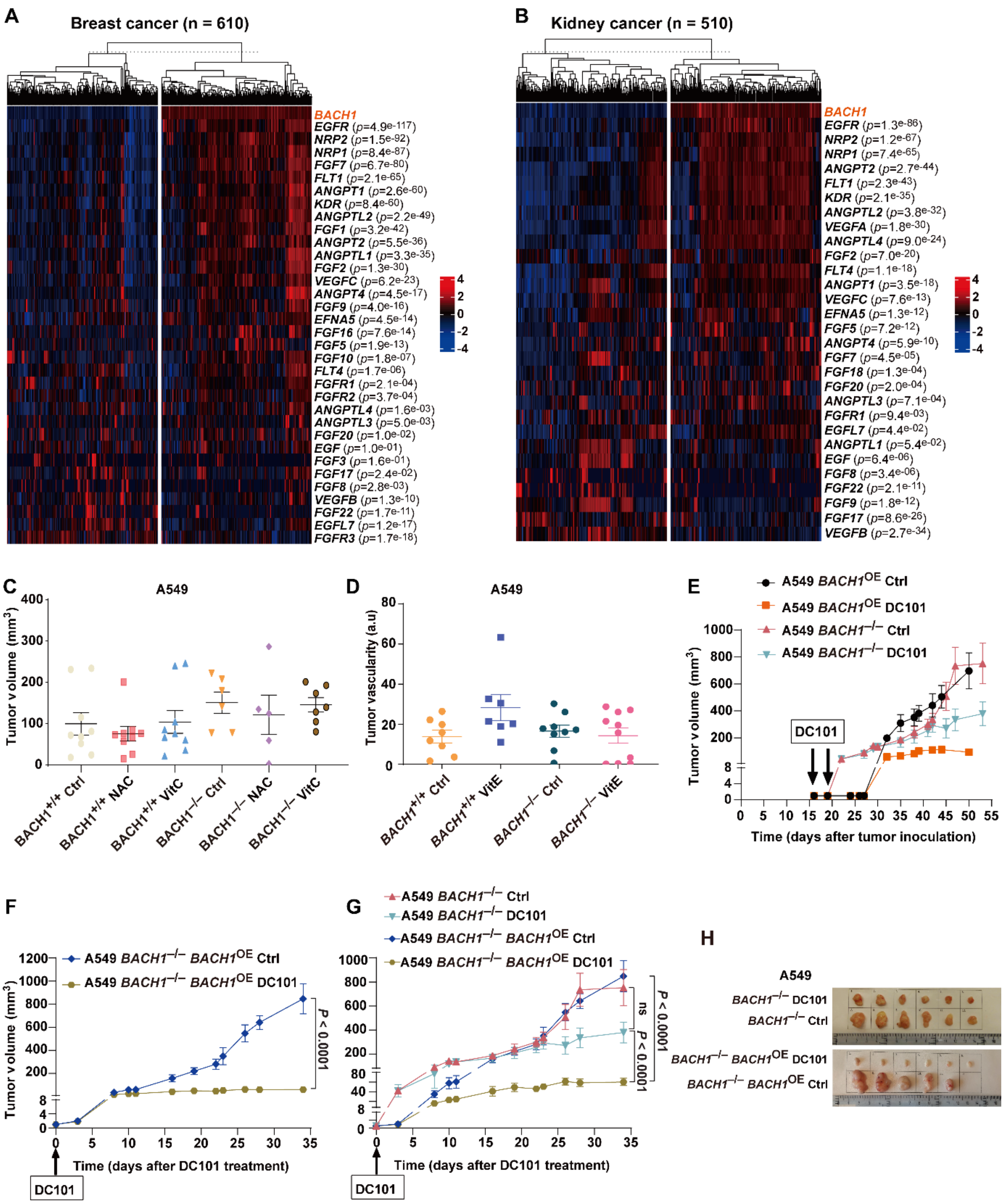

Supplemental Figure 9. BACH1 expression correlates with angiogenesis gene expression in human breast and kidney cancer samples and increases the response to anti-VEGF therapy in xenografts. (**A**) Heat maps showing TCGA breast cancer cases with low (left) and high (right) *BACH1* expression. Angiogenic genes whose expression differed significantly between the two groups are listed on the right along with p value for the correlation with *BACH1* expression. (**B**) TCGA kidney cancer cases analyzed as in panel A. (**C**) Tumor volume (percent of Ctrl) at endpoint from the experiment shown in Figure 7A and 7B. (**D**) Tumor vascularity (peak enhancement) in *NSG* mice injected s.c. with 5 × 10^5 *BACH1*$^{+/+}$ or *BACH1*$^{-/-}$ A549 cells and administrated with 0.5 g/kg VitE in the chow (n = 7 and 10 for +/+ and –/–, respectively)

or received control food (n = 8 and 9, respectively) for 7 weeks. (**E**) Same experiment as in Figure 7D and 7E, but with the time from tumor cell injection to the end of the experiment shown on the x-axis (Figure 7D and 7E shows time from start of DC101 injections): note that *BACH1*$^{-/-}$ cells produced palpable tumors prompting injection with anti-VEGFR2 antibodies (DC101) three days after the *BACH1*^{OE} cells; moreover, the mice carrying *BACH1*$^{-/-}$ tumors reached the humane endpoint for euthanasia three days after mice with *BACH1*^{OE} tumors. (**F–H**) Tumor growth in *NSG* mice injected s.c. with 5 × 10^5 *BACH1*$^{-/-}$ *BACH1*^{OE} (F and H) A549 cells. When tumors were palpable, the mice were injected i.p. with PBS (n = 5) and 40 mg/kg DC101 (n = 6) three times a week for five weeks. Tumors were measured three to five times per week. (**G**) Curves from panels Supplementary 9F and Figure 4H shown in the same graph. Data are mean and SEM. Statistics: Two-way ANOVA with Sidak's post-hoc test for multiple comparisons (panel G). Panel H (upper image) was previously shown in Figure 7C (lower image).

Supplemental Table 1. Direct BACH1 target genes in human lung tumor cells

Gene	Function	Effect
GLUT1		
GLUT3		
PFKP		
PFKFB2		
PFKFB3	Glycolysis	Activation
PKM		
PGK1		
PDK1		
LDHA		
GAPDH		
HK2		
VEGFA		
VEGFC		
VEGFD		
VEGFR1		
VEGFR2		
VEGFR3		
NRP2		
FGF2		
FGF9	Angiogenesis	Activation
FGFR1		
FGFR2		
FGFR3		
FGFR4		
ANGPT2		
ANGPTL1		
ANGPTL4		
EGF		
EGFL7		
EFNA5		

Supplemental Table 1. Direct BACH1 target genes and their regulation in human lung cancer cells. Total list of genes identified and validated as BACH1 targets in CUT&Tag and RT-qPCR experiments.

Supplemental Table 2. Characteristics of the analyzed patients with KRAS mutation

**Characteristics**		**KRAS mutation (n=20)**
**Sex, n(%)**	Male	14(70.0%)
	Female	6(30.0%)
**Age, median (min, max), year**		64.5(54-79)
**Tumor location, n(%)**	Upper lobe of left lung	3(15.0%)
	Lower lobe of left lung	8(40.0%)
	Upper lobe of left lung	4(20.0%)
	Middle lobe of left lung	2(10.0%)
	Lower lobe of left lung	3(15.0%)
**Differentiation, n(%)**	Well	2(10.0%)
	Moderate	6(30.0%)
	Poor	12(60.0%)
**T stage, n(%)**	T1	7(35.0%)
	T2	11(55.0%)
	T3	1(5.0%)
	T4	1(5.0%)
**N stage, n(%)**	N0	11(55.0%)
	N1	3(15.0%)
	N2	6(30.0%)
**M stage, n(%)**	M0	16(80.0%)
	M1	4(20.0%)
**cTNM stage*, n(%)**	I	8(40.0%)
	II	1(5.0%)
	III	7(35.0%)
	IV	4(20.0%)

* 8th AJCC staging.

Categorical variables were expressed as frequencies (n) and proportions (%).

Continuous variables were expressed as frequencies (n) and proportions (%).

Supplemental Table 2. Characteristics of patients and tumors.
